# 3MSF: A Multi-Modal Adaptation of the 6TiSCH Minimal Scheduling Function for the Industrial IoT

**DOI:** 10.3390/s24082414

**Published:** 2024-04-10

**Authors:** Robbe Elsas, Dries Van Leemput, Jeroen Hoebeke, Eli De Poorter

**Affiliations:** IDLab, Department of Information Technology, Ghent University—imec, 9052 Ghent, Belgium; dries.vanleemput@ugent.be (D.V.L.); jeroen.hoebeke@ugent.be (J.H.); eli.depoorter@ugent.be (E.D.P.)

**Keywords:** 6TiSCH, 6top, IEEE 802.15.4, IIoT, IPv6, multi-hop, multi-modal, RPL

## Abstract

Although wireless devices continuously gain communication capabilities, even state-of-the-art Industrial Internet of Things (IIoT) architectures, such as Internet Protocol version 6 over the Time-Slotted Channel Hopping (TSCH) mode of IEEE 802.15.4 (6TiSCH), continue to use network-wide, fixed link configurations. This presents a missed opportunity to (1) forego the need for rigorous manual setup of new deployments; and (2) provide full coverage of particularly heterogeneous and/or dynamic industrial sites. As such, we devised the Multi-Modal Minimal Scheduling Function (3MSF) for the TSCH link layer, which, combined with previous work on the routing layer, results in a 6TiSCH architecture able to dynamically exploit modern multi-modal hardware on a per-link basis through variable-duration timeslots, simultaneous transmission, and routing metric normalization. This paper describes, in great detail, its design and discusses the rationale behind every choice made. Finally, we evaluate three basic scenarios through simulations, showcasing both the functionality and flexibility of our 6TiSCH implementation.

## 1. Introduction

Industrial Internet of Things (IIoT) devices increasingly possess multiple radio interfaces, each supporting multiple physical layers (PHYs) [1]. Nonetheless, higher protocol layers have not co-evolved to exploit these multi-modal capabilities, generally resulting in a single static link configuration being used throughout the network. Limiting all network devices to the same, statically configured communication mode (i.e., a given configuration of a given PHY) leads to sub-optimal performance, especially in dynamic environments. Additionally, this strategy often necessitates extensive manual setup when deploying IIoT networks across different sites. Moreover, extremely heterogeneous environments such as petrochemical installations may not even be fully serviceable with a single communication mode, leading to gaps in network coverage. As such, a network architecture that dynamically engages the multi-modal capabilities of modern IIoT devices on a per-link basis is needed to meet the demands of diverse operational landscapes.

Over the past decade, a distinct subset of open protocol layers defined by the Internet Engineering Task Force (IETF) for use with the IEEE 802.15.4 PHY and Medium Access Control (MAC) layers [2] has become synonymous with IIoT-related research. Conceivably, this “IETF stack” partially owes its popularity to the promise of seamless integration into existing Internet Protocol (IP) infrastructure. However, besides the convenience of end-to-end IP connectivity, it also offers technical benefits specific to industrial applications, especially since the IEEE 802.15.4e amendment [3] introduced simultaneous Time-Division Multiple Access (TDMA) and Frequency-Division Multiple Access (FDMA) through the Time-Slotted Channel Hopping (TSCH) MAC mode, which has recently resulted in several IETF Requests For Comments (RFCs) related to an IP version 6 (IPv6) over the TSCH mode of IEEE 802.15.4 (6TiSCH) architecture [4,5].

Nonetheless, 6TiSCH (the latest iteration of the IETF stack) still suffers from its MAC and routing layers typically not being configured to accommodate multi-modal operation. This is unfortunate, especially since both TSCH and the Routing Protocol for Low-Power and Lossy Networks (LLNs) (RPL) [6] (the 6TiSCH routing layer) offer considerable implementational flexibility through TSCH Scheduling Functions (SFs) and RPL Objective Functions (OFs), respectively.

To overcome these limitations, we designed the Multi-Modal Minimal Scheduling Function (3MSF), which is partially based on (1) the 6TiSCH Minimal Scheduling Function (MSF) [7]; (2) a state-of-the-art solution from literature; and (3) design considerations for a multi-modal 6TiSCH architecture we identified in prior work [1]. In conjunction with a slightly modified version of an RPL OF previously designed for a non-TSCH-based version of the IETF stack [8], this constitutes our attempt at a multi-modal 6TiSCH architecture capable of dynamic link reconfiguration. While (few) others (see Section 3) have made similar attempts, our solution is the first to fully harness the potential of modern IIoT devices through a combination of, among other things, variable-duration timeslots, simultaneous transmission, and allowing all management traffic to be sent on more than one communication mode, all while staying compliant with 6TiSCH.

The remainder of this paper is structured as follows. We first explain the basic working principles of 6TiSCH and outline several considerations for multi-modal operation in Section 2 to better understand the related work in Section 3. Next, we break down the main concepts behind our new TSCH SF in Section 4 before describing a set of well-chosen simulation scenarios and examining their results in Section 5. Finally, Section 6 summarizes our findings.

## 2. IPv6 over the TSCH Mode of IEEE 802.15.4

Supporting multi-modal operation affects all layers that derive (part of) their functionality from link-layer operation. Besides always affecting the link layer itself, it can also affect the routing layer, particularly if routing tables are (indirectly) based on, e.g., statistics a node infers about its connections to neighboring devices.

6TiSCH is no different; its link and routing layers require significant modification when multi-modal operation is the goal. However, 6TiSCH is uniquely suited for this task as its link and routing layers are particularly flexible. In any case, we first explain the basic working principles of 6TiSCH with a focus on the MAC and, to a lesser extent, routing layers in Section 2.1. Second, Section 2.2 outlines several considerations for applying multi-modal operation to 6TiSCH in particular.

### 2.1. 6TiSCH Architecture Basics

**Figure 1:**
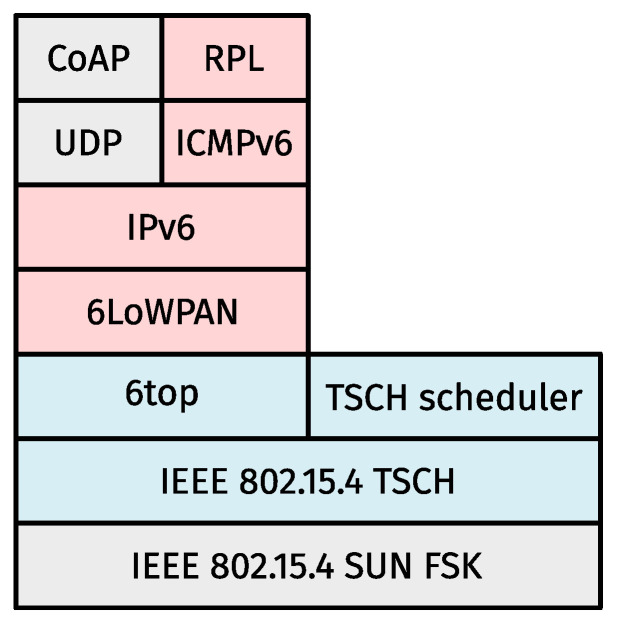
Example of a common implementation of the Internet Engineering Task Force (IETF) Internet Protocol version 6 (IPv6) over the Time-Slotted Channel Hopping (TSCH) mode of IEEE 802.15.4 (6TiSCH) protocol stack. Network-layer functionality is indicated in red, whereas link-layer functionality is marked blue. Reprinted from [1] with permission from the authors.

Figure 1 depicts a high-level representation of a common 6TiSCH stack. The IEEE 802.15.4 multi-layer standard defines an abundance of PHYs. In addition, TSCH deliberately leaves its most important component (i.e., the SF) open for implementation. Similarly, RPL does not rigidly define next-hop determination. That is, as long as some universal rules for building routing tables are adhered to, anything goes. While this inherent flexibility makes 6TiSCH a powerful solution for demanding use cases, the sheer number of configuration permutations makes it a prime candidate to fall victim to “choice overload” [9]. As the remainder of this paper focuses on the link and, to a lesser extent, routing layers, Section 2.1.1 and Section 2.1.2 give a quick primer on how these respective layers would operate ordinarily (i.e., if all nodes were statically configured to use a single PHY) when applied to Figure 1.

#### 2.1.1. The Routing Layer

**Figure 2:**
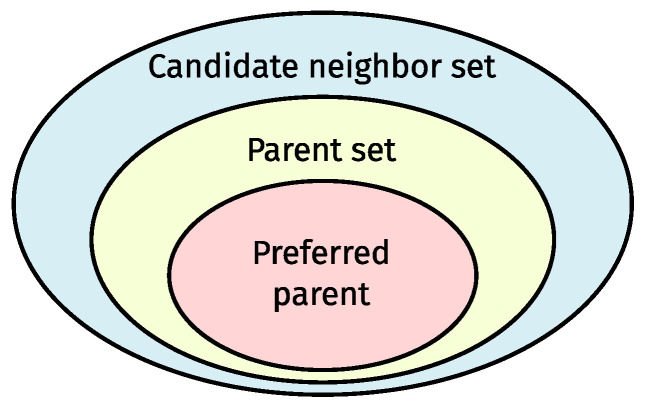
Connection between the three Routing Protocol for Low-power and Lossy Networks (LLNs) (RPL) neighbor sets maintained by each non-root node. The formation of these sets is prescribed by an RPL Objective Function (OF). Reprinted from [8] with permission from the authors.

RPL is 6TiSCH’s routing protocol. It functions as a distance-vector routing protocol, constructing a hierarchical/tree-shaped routing structure named a Destination-Oriented Directed Acyclic Graph (DODAG) that converges towards a root node. Within the DODAG, each non-root node (theoretically speaking) maintains three distinct neighbor sets, as depicted in Figure 2: (1) the candidate neighbor set; (2) the parent set; and (3) the preferred parent, which is the default next hop towards the root.

These sets are managed through control messages known as DODAG Information Objects (DIOs). More specifically, a DIO advertises a node’s rank, which is the distance-vector element of RPL, i.e., “a scalar representation of the location or radius of a node within a DODAG Version” [6] (p. 20). Following an end-to-end path to the DODAG root, node ranks are required to decrease in a monotonic fashion.

Within a DODAG, the flow of traffic is primarily Multi-Point to Point (MP2P), making the mechanisms by which an upward routing topology is formed and maintained critical. For instance, when an RPL node wants to join a DODAG shortly after booting and receiving a DIO, it needs to attach itself to the DODAG first and acquire a rank before it may initiate self-advertising and potentially become a parent. A node is attached once it has a non-empty parent set and determines the rank to advertise (towards potential children) in forthcoming DIOs either during or after filtering the candidate neighbor set down to a suitable parent set and choosing a preferred parent from it.

Discovering upward routes and forming/maintaining an upward routing topology are both controlled by an OF. As per RFC 6550 [6] (p. 67), “the candidate neighbor set is a subset of the nodes that can be reached via link-local multicast. The selection of this set is implementation and OF-dependent”. Additionally, the OF largely dictates how a parent set should be formed from the set of candidate neighbors. That being said, OF-agnostic rules do apply. The primary tasks of the OF are to select a preferred parent and compute a rank to advertise. Both usually involve a routing metric [10]. Whether or not rank computation is part of parent set formation and preferred parent selection can differ between OFs. We kindly refer the reader to prior work [8] for a more detailed explanation of RPL and the role of OFs.

#### 2.1.2. The Link Layer

TSCH is the MAC protocol of 6TiSCH. TSCH requires nodes to synchronize with a slotframe that repeats periodically, every sub-division of which, referred to as a “timeslot”, must accommodate the transmission of a data frame followed by a corresponding acknowledgement. The Absolute Slot Number (ASN) counts the timeslots that have passed since the network’s inception, i.e., since the root started advertising the DODAG. TSCH also employs multi-channel communication. To this extent, the “hopping sequence” contains all eligible channels. The channel on which to communicate can always be calculated using (1); hence, the definition of a TSCH link as “the pairwise assignment of a directed communication between devices … in a given timeslot on a given [channel offset]” [2] (p. 70).

(1) CH=HS[(ASN+CO)%|HS|]

where:
CHthe frequency channel to communicate on;HSa finite ordered (≠sorted) list (≠set) of frequency channels to communicate on, i.e., the “hopping sequence”;ASNthe absolute slot number of the current timeslot;COthe current channel offset, as determined by the TSCH scheduler;|HS|the length of the hopping sequence.

Consequently, if distinct pairs of nodes use different channel offsets, they can communicate during the same timeslot. Should the reader be confused about slotframes, timeslots, and the like, we highly recommend reading Appendix A.

The “link schedule”—the allocation of TSCH links to nodes and/or node pairs—is central to TSCH. IEEE 802.15.4 leaves the choice of a scheduling algorithm to the implementer. As a result, many TSCH schedulers exist, all of which can generally be categorized as either (1) centralized; (2) decentralized; or (3) autonomous.

Choosing/designing an SF involves a trade-off between several factors, which are beyond the scope of this paper. For this work, we considered the decentralized approach most appropriate because it offers improved flexibility compared to an autonomous approach, without incurring the full overhead penalty of a centralized alternative.

With a decentralized SF, scheduling decisions are made locally by individual nodes rather than relying on a central coordinator. Each node maintains its own (local) schedule of transmit/receive links towards its neighbors based on its communication requirements and information gathered from those neighbors. As such, decentralized SFs require a way to communicate these requirements with neighboring nodes. Conveniently, there is a well-defined set of decentralized scheduling semantics with the specific purpose of building/maintaining a local schedule. These semantics are called the 6TiSCH Operation Sublayer (6top) Protocol (6P) [11], and 6top effectively forms a layer in between the TSCH MAC and higher protocol layers. All 6P-related information is carried in a sub-type of the IETF Informational Element (IE) [12] called the 6top sub-IE [11] (Section 3.2.1), typically in otherwise empty data frames.

Arguably the most popular/well-defined SF that uses 6P is MSF [7], which breaks traffic down into three planes and ties a slotframe to every plane (i.e., the minimal, autonomous, and negotiated slotframes, respectively) all of which align 100%. As with all schedulers using multiple slotframes, they are merged in a “rolling” fashion according to TSCH precedence rules [2] (Section 6.2.6.4). More specifically, when determining the next link to use, if there are overlapping cells in different slotframes, the cells housing a transmit (Tx) link have precedence. If there is still ambiguity after that, the cell from the slotframe with the lowest handle has precedence. Overlap is always meant in terms of ASN only. That is, if two cells occur at the same ASN (which, for MSF, means they will have the same slot offset), they overlap, even if they have different channel offsets. For the remainder of this paper, we understand the term(s) cell (and supercell, see Section 4) to also mean link unless noted otherwise.

MSF’s minimal slotframe, which it borrows from RFC 8180 [4], consists of a single cell with slot and channel offsets of zero. This minimal cell is a shared advertising cell and can be used to transmit/receive all frame types, but it is mostly used for broadcast traffic, including TSCH Enhanced Beacons (EBs), as broadcast traffic is not allowed on cells of other slotframes. MSF prohibits overlap with the minimal cell.

In the autonomous slotframe, a node permanently installs a single receive (Rx) cell, the slot- and channel offsets of which are hashes of its own link-layer address. Under certain conditions, a node may also add (and later remove) a shared Tx cell to a neighbor using the same hashing functions to derive the cell’s offsets from its neighbor’s link-layer address. The cells within this slotframe are referred to as autonomous Rx and Tx cells, respectively. The autonomous Rx cell can only overlap with autonomous Tx cells, whereas autonomous Tx cells can overlap with all types of cells except the minimal cell.

As long as a node has at least one negotiated Tx cell towards a neighbor, having an autonomous Tx cell towards it is forbidden. In addition, an autonomous Tx cell must be removed when there are no more packets for the corresponding neighbor. MSF is designed to work with RPL in the sense that a node must add at least one negotiated Tx cell towards a parent when it becomes preferred and must remove all negotiated cells (Tx or Rx) towards a parent as soon as it is no longer preferred.

Negotiated cells are exclusively managed through a set of two-step 6P transactions, all of which (except CLEAR transactions) can only be initiated by a node sending a 6P request to its preferred parent. That is, when a node switches its preferred parent, it initiates the appropriate 6P transactions (at appropriate times), and it does so using autonomous cells (applies to both the old and new preferred parent) until it has its first negotiated Tx cell (applies to the new preferred parent only), after which all unicast traffic to the new preferred parent is sent across the negotiated Tx cells the node has added (it may have negotiated multiple Tx cells at once), including further 6P traffic. When, which type of, and how many negotiated cells are added and/or removed is subject to many rules (see RFC 9033 [7]). Negotiated cells are not shared.

### 2.2. Considerations for Multi-Modal Scheduling

**Figure 3:**
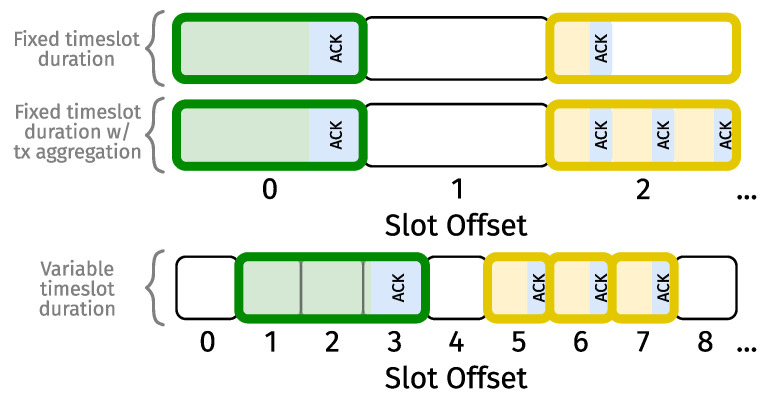
Abstract representation of multi-mode TSCH slotframes using fixed- or variable-duration timeslots, respectively, in the time dimension only. Each coloured box corresponds to one (virtual) timeslot, with a dedicated colour associated with each communication mode (each having a specific data rate). Note that there are other ways to aggregate consecutive transmissions within a fixed-duration timeslot. Reprinted from [1] with permission from the authors.

Since different communication modes (i.e., specific configurations of given PHYs) may have different data rates, an obvious consideration is the duration of timeslots. More specifically, do we use timeslots of the same (fixed) duration for all modes, or do we vary their duration corresponding to the data rate? In comparison to fixed duration, variable duration enhances schedule efficiency, resulting in increased throughput and/or decreased end-to-end latency. That being said, TSCH channel hopping (1) calls for (1) the ASN to be based on identical time boundaries throughout the network (±drift); and (2) a transmission (+potential acknowledgement) to respect said boundaries. If slots are of variable duration, there is only one way (known to us) to meet the first condition, i.e., by grouping together consecutive timeslots of equal duration. Unfortunately, this approach violates the second condition, although this would seemingly be a non-issue if the SF knew about timeslot durations. Fixed timeslot duration, on the other hand, simply follows the slowest communication mode. Through a one-dimensional slotframe representation (see Appendix A), Figure 3 illustrates the difference between using fixed- or variable-duration timeslots.

For the remainder of this paper, we refer to timeslots of variable duration as “superslots” and their constituent slots of unit duration as “subslots”, assuming a one-dimensional slotframe representation. In two dimensions, this concept translates to “supercells” and “subcells”, respectively. The subcells of a supercell must be consecutive. In the time dimension, this means they must occupy consecutive slot offsets. In addition, assuming the frequency channel (≠ channel offset) cannot change mid-transmission, subcells must also be organized accordingly in terms of channel offsets. After all, since the ASN increments for consecutive slot offsets, the frequency channel corresponding to those slot offsets follows (1). As such, the slot and channel offsets required for the subcells of a supercell to be truly consecutive are determined using (2) and (3).

(2) $$T_{a,b}=\left\{n\in N:0\leq a\leq n\leq b<|SO|\right\}$$

(3) $$CO_{SO}=\left\{\begin{array}{cc} \left\{\begin{array}{cc} hash(M\;AC_{dest}) & \mathrm{for}\;\mathrm{Auto}-\mathrm{TX}\;\mathrm{cells}\\ hash(MAC_{src}) & \mathrm{for}\;\mathrm{Auto}-\mathrm{RX}\;\mathrm{cells}\\ rand(0,|CO|-1) & \mathrm{for}\;\mathrm{Negotiated}\;\mathrm{cells} \end{array}\right. & \mathrm{when}\;SO=a\\ \left(\left|CO\right|-1+CO_{SO-1}\right)\;\%\;\left|CO\right| & \forall \;SO\in T_{a+1,b} \end{array}\right.$$

where:
$T_{a,b}$the set of consecutive slot offsets in the supercell starting at slot offset *a* and ending after slot offset *b*;|SO|the number of slot offsets in a slotframe (starting from 0), i.e., the “slotframe length”;SOa slot offset expressing a discrete time position relative to the start of the slotframe;$CO_{SO}$the channel offset at a given slot offset in the slotframe;|CO|the number of channel offsets in a slotframe (starting from 0).

Besides timeslot duration, determining which communication modes to use for management traffic is another important consideration. Fully or even partially restricting management traffic to one fixed communication mode typically necessitates elaborate in situ experiments and/or simulations. Moreover, if the existence or continuity of a network hinges on only one mode of communication, the advantages of said network being multi-modal are essentially restricted to enhancing its performance in settings where single-mode technologies could already achieve ubiquitous connectivity. Such approaches fail to capitalize on a key advantage of multi-modal IIoT—specifically, the greatly enhanced probability of achieving full coverage throughout all regions of a single deployment, even in the absence of prior knowledge and with highly localized, time-varying conditions being probable.

Finally, with synchronized MAC protocols like TSCH, you cannot simply tag packets with the communication mode to use for transmission. After all, devices can have multiple modes per interface, and although it is theoretically possible to simultaneously transmit and/or receive on modes belonging to different interfaces (provided they operate in different frequency ranges or the modes are otherwise orthogonal), the same is not generally true for communication modes belonging to the same interface due to hardware constraints. Hence, with TSCH specifically, it is better to tag cells with the communication mode(s) to use within them so that receiving nodes know a priori which communication mode(s) to listen to when waking up for an Rx cell. Put differently, if you wanted to tag packets, a receiving node would need to be able to listen on all interface-modes at once. Since this either imposes unreasonable hardware requirements on network devices or unnecessarily limits them to one communication mode per interface, tagging cells is a better approach. Moreover, even if nodes could listen to all possible communication modes at once, including those belonging to the same interface, doing so in every cell would consume significantly more energy.

## 3. Related Work

Other researchers have also contemplated choosing between either fixed- or variable-duration timeslots. For example, Brachmann et al. [13] envisioned using multi-mode TSCH slotframes to address application-specific performance requirements, mitigate the impact of interference, etc. For this purpose, the authors put forward two design strategies. Disregarding the authors’ fixed-duration solution (which only served to characterize the medium), their variable-duration alternative uses a single low-rate PHY for TSCH beacons while sending all other traffic across a different/high-rate PHY. In other words, rather than being allowed to switch between communication modes (at run-time) on a per-link basis, devices must do so based on traffic type instead.

Van Leemput et al. [14] demonstrated that fixed-duration timeslots can achieve considerable throughput. Hence, they saw value in the reduced implementational complexity of a fixed-duration approach and enabled the aggregation of multiple transmissions in one timeslot. The authors proposed two strategies for aggregating transmissions in unicast links, i.e., sending an acknowledgement in response to (1) individual unicast packets; or (2) a burst of unicast packets.

Both of the authors’ strategies are based on an autonomous SF called Orchestra [15], employing a single low-rate PHY for broadcast and beacon slots while enabling unicast slots to utilize either the low-rate or a different high-rate PHY. As the duration of a timeslot allows for one low-rate transmission, aggregating transmissions is only allowed in unicast slots. From a throughput perspective, transmission aggregation is most advantageous for unicast traffic anyway.

Van Leemput et al. [14] explicitly reconfigured unicast links to use a certain PHY. Beginning from the PHY with the lowest data rate, a link’s receiving endpoint assesses the quality of the link upon packet reception and, through a rate-limiting mechanism, determines whether switching to a PHY with a higher data rate (i.e., beginning with the next occurrence of a timeslot for the link) is appropriate or not. The decision is subsequently sent to the transmitting endpoint by repurposing bits within the returned acknowledgement(s). To prevent link configuration inconsistencies (and by extension, dropped packets) due to a lost acknowledgement, if the transmitter fails to receive an acknowledgement for four Tx attempts in a row (on a unicast link), it will automatically transition to the PHY with the next highest data rate. Proper configuration of the reconfiguration rate-limiting mechanism is essential, as cycling through PHYs is costly.

**Figure 4:**
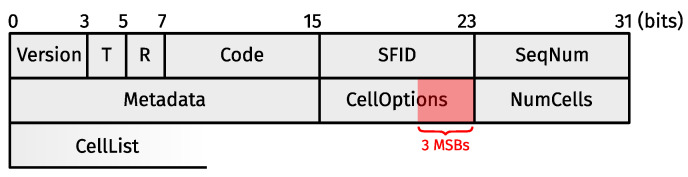
Format of a 6TiSCH Operation Sublayer (6top) Protocol (6P) ADD request. The three Most Significant Bits (MSBs) of the CellOptions field indicate the communication mode to use in the supercells/subcells proposed by the CellList.

Rady et al. [16] based their variable-duration slot approach on MSF (see Section 2.1.2), which required several adaptations. Firstly, the minimal slotframe contains a minimal supercell for each unique communication mode. Nodes use the TSCH Slotframe and Link Informational Element (IE) [2] (Figure 7-52) carried in TSCH EBs to reserve groupings of subcells with consecutive slot offsets (starting from slot offset 0). Each subcell grouping forms the minimal supercell for a given mode. Additionally, the three Most Significant Bits (MSBs) of a LinkOptions field [2] (Figure 7-55), which are ordinarily reserved for future use, now indicate the communication mode to use in minimal supercells/subcells. Secondly, a node still has at most one autonomous Tx supercell towards any of its neighboring devices (≠RPL neighbors, in this case), and all autonomous supercells use the same communication mode. Thirdly, the authors proposed using the three MSBs of the CellOptions [11] (Section 3.2.3) field present in certain 6P requests, such as the one depicted in Figure 4, to indicate the communication mode to use in the negotiated supercells that are the subject of said 6P management traffic.

Rady et al. [16] identified a similar need (see Section 2.2) for annotating cells with communication modes. However, they tag cells with an identifier corresponding to a single communication mode, meaning simultaneous transmission on modes of different interfaces is not possible. This works for their design because they define every communication mode to be an independent RPL neighbor (devices remain the addressable entities on the link and routing layers) and assume the use of an Rx-based routing metric. More specifically, their solution does not work with exclusively Tx-based routing metrics because there is no way to keep fresh metrics for all RPL neighbors with autonomous supercells always using the same communication mode and every communication mode being an independent RPL neighbor (like most 6TiSCH implementations, they assume a node has one preferred parent at a time), whereas with an Rx-based routing metric, the broadcast packets received on the minimal supercells would suffice. The problem is that Rx-based routing metrics are often rather simplistic.

Moreover, autonomous supercells are quite important for network performance, as they typically service a large part of 6P traffic, especially in the downstream direction of networks that are, after all, mostly MP2P, meaning negotiated Rx supercells towards preferred parents are not common (nor are they mandatory). Put differently, limiting autonomous supercells to a single communication mode is a huge potential weakness (and we have not even touched yet on their importance when switching to a new preferred parent). Having multiple autonomous supercells towards the same device, each using a single communication mode, is also not an option because that would be extremely inefficient, which further highlights the missed opportunity for simultaneous transmission on communication modes of interfaces that operate in different frequency ranges or are otherwise orthogonal.

Recently, Bunn et al. [17] proposed enabling simultaneous multi-band operation for 6TiSCH by adding a secondary radio interface to devices (one fixed mode per interface), building an independent routing topology for each interface, and sending a duplicate of the same packet in every topology. While this reduces routing layer complexity, building a separate DODAG for each communication mode without the possibility of switching packets between them implies a reduced capability to handle heterogeneous environments compared to single-topology solutions. After all, as a given copy of a packet can only progress within its DODAG, each possible destination must be reachable across at least one path of which each link uses the same communication mode. That is, if you want to achieve full coverage. Additionally, sending a duplicate of each message for every communication mode, combined with the added management traffic associated with maintaining multiple independent routing topologies, means that a multi-topology strategy is less energy efficient. As a counter-argument, the authors claimed that single-topology solutions are inherently non-compliant with 6TiSCH, which is factually incorrect. Presumably, they failed to recognize that 6TiSCH does not mandate the use of any specific RPL OF.

In summary, Table 1 gives an overview of the TSCH-based solutions for multi-modal IIoT discussed in this section and how they differ (mainly) in terms of the considerations discussed in Section 2.2. Note that the work of Bunn et al. [17] is not included in this table, as it constitutes a fundamentally different (and arguably sub-optimal) approach.

## 4. The Multi-Modal Minimal Scheduling Function

Based on MSF, the related works in Section 3, and prior work [1,8], we designed a multi-modal variant of the 6TiSCH stack, as depicted in Figure 1. Central to this effort is our new SF (i.e., 3MSF), which uses supercells and hence constitutes a variable-duration slot approach. This section outlines the ground rules for 3MSF. More specifically, Section 4.1 describes how our perceived need for simultaneous Tx requires an abstracted way of tagging cells. Section 4.2 gives an overview of the 3MSF slotframes and how they are different from the solutions that inspired them. Next, Section 4.3 discusses how timeslot timings relate to supercell lengths, followed by a description of how supercells mandate a new way to determine the next link to wake up for in Section 4.4, as well as an explanation of all the different 6P transactions 3MSF uses (and when it does) in Section 4.5. Finally, Section 4.6 discusses the use of routing metrics, a topic that is more indirectly related to 3MSF but played a major role in its design nonetheless.

### 4.1. TSCH Mode Abstraction

As we wanted to use a Parent-Oriented (PO) approach (meaning devices are RPL neighbors) based on prior work [1,8] combined with a Tx-based routing metric (see Section 4.6), we needed a way to keep fresh inferred metrics without crowding the schedule. Hence, we devised an abstraction called “TSCH modes”. A TSCH mode uniquely identifies which communication mode(s) to use in a (super)cell, and (potentially) when to do so. Firstly, we defined a unique non-simultaneous TSCH mode for each unique communication mode. In a supercell tagged with a non-simultaneous TSCH mode, transmission can only occur on the corresponding communication mode. Through a process we elaborate on in Section 4.3, one can determine the number of consecutive subcells required for a supercell tagged with a non-simultaneous TSCH mode. The timeslot timings (see [2] (Section 6.5.4.2)) to use follow from this supercell length.

Additionally, we defined a group of simultaneous TSCH modes. During simultaneous transmission, an identical copy of a packet (including MAC sequence number) is sent on all (available) communication modes pointed to by the simultaneous TSCH mode. The length of a supercell tagged with a simultaneous TSCH mode equates to the longest supercell length that would ordinarily be used for any non-simultaneous TSCH mode corresponding to the communication modes it points to. For example, if the non-simultaneous TSCH modes corresponding to its underlying communication modes require a supercell of length two and three, then a supercell tagged with a simultaneous TSCH mode has a supercell length of three. Simultaneous transmissions must adhere to the timeslot timings required for this longest underlying supercell length.

Alternating TSCH mode is a special simultaneous mode that almost entirely evaluates to one of the other simultaneous TSCH modes (including its timings), depending on the starting ASN and the length of the slotframe (all 3MSF slotframes are of equal length, see Section 4.2). The only difference is that the length of a supercell tagged with alternating mode is fixed to the longest required supercell length of any of the simultaneous TSCH modes to which it can evaluate. Alternating TSCH mode provides a means of cycling through all possible interface-modes towards a neighbor while occupying a minimal amount of time-frequency space.

The TSCH mode abstraction uses a 3-bit representation, as we use the same (currently reserved) protocol bits used by Rady et al. [16], i.e., the three MSBs of the CellOptions field contained in certain 6P requests (see Section 4.5) and the three MSBs of the Link Options fields (one per minimal supercell) contained in TSCH EBs. Since the amount of information one can encode with 3 bits is limited, we made (reasonable) assumptions about the hardware capabilities of an average 6TiSCH node. More specifically, we assumed all devices to have at most two interfaces (which operate in different frequency ranges or the communication modes of which are otherwise orthogonal between them), each supporting at most two unique communication modes. In addition, all network devices must either own the same set of interface-modes or otherwise be aware of the superset of interface-modes that exist throughout the network (presumably through some mechanism that is beyond the scope of this paper).

Barring two special values, the 3-bit TSCH mode encoding follows an *m.ii* dot notation format (MSB-first), where ii denotes the index of an interface in an ordered list of interfaces known to all nodes and *m* denotes the index of the communication mode in one of two ordered lists (i.e., the one corresponding to the interface with index ii), both of which are known to all nodes. Table 2 lists all TSCH mode encodings.

### 4.2. Slotframe Structure

**Figure 5:**
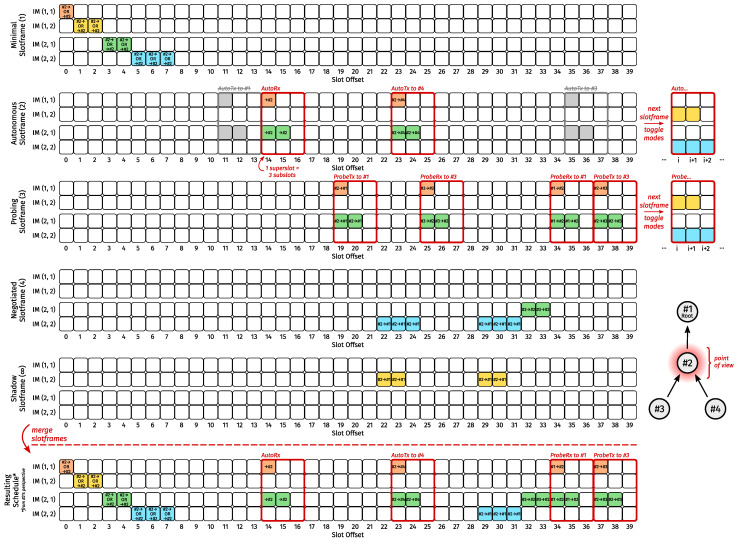
Example of the slotframe structure of the Multi-Modal Minimal Scheduling Function (3MSF). As 3MSF is a distributed scheduler, the contents of the slotframes shown constitute the schedule as seen by a single node (i.e., #2) in a small example network. Black arrows indicate child–parent relationships.

For 3MSF, we maintained (in spirit) the three slotframes of Rady et al. [16] (and MSF) and added two more. The minimal slotframe is virtually identical. As shown in Figure 5, it contains a minimal supercell for every unique interface-mode. The string of minimal supercells starts at a slot offset of zero and spans exactly as many consecutive subcells (see Section 2.2) as needed to accommodate one supercell per unique interface-mode without the minimal supercells overlapping with one another. When a new slotframe comes around and it is time to advertise the TSCH network, an identical EB must be broadcast on each consecutive minimal supercell of said slotframe, with no retransmissions allowed. This could be achieved by, for example, having a separate buffer (i.e., from the broadcast buffer) for EBs, giving absolute priority to EBs in minimal supercells, and simply adding as many EBs to the EB buffer as there are minimal supercells.

At first glance, the negotiated slotframe also incurs minimal changes from Rady et al. [16] (and MSF) in the sense that, e.g., adding at least one negotiated Tx supercell towards an RPL parent when it becomes preferred remains mandatory. However, we consider devices, and not individual communication modes, to be independent RPL neighbors. Therefore, the cost associated with each communication mode is no longer automatically accounted for through routing layer operations alone. Negotiated supercells can only overlap with autonomous Tx supercells and certain so-called “shadow” supercells (more about that later). All negotiated supercells towards a given neighbor can only be tagged with a single non-simultaneous TSCH mode at a time.

Our autonomous slotframe uses the alternating TSCH mode (see Section 4.1), mostly to maintain up-to-date inferred metrics for all interface-modes of each neighbor towards whom a node can (still) use an autonomous Tx supercell. Since autonomous supercells always occupy the same number of subcells, we slightly modified the hashing function to ensure they fall within predetermined bins, meaning overlapping autonomous supercells start and end at the same slot offsets.

With MSF, a node cannot use an autonomous Tx cell towards a neighbor to which it already has a negotiated Tx cell. However, in our case, if we have negotiated Tx supercells towards a given neighbor, we also require at least one Tx supercell tagged with the alternating TSCH mode towards the same neighbor. Otherwise, we could not maintain fresh inferred metrics towards it.

The only way to maintain fresh inferred metrics and consistent MAC sequence numbers is to allow non-probe (see Section 4.6) unicast packets on both kinds of supercells. That is, once we have attempted transmission of a unicast packet with a given MAC sequence number towards a given neighbor, we cannot send it a unicast packet with a lower sequence number before a certain (typically quite long) lifetime expires to maintain sequence number consistency. Therefore, once we attempt transmitting a unicast packet (and its MAC sequence number is “on-air”), it must be dequeued before the next unicast packet (i.e., with a consecutive sequence number) can be transmitted to the same neighbor. Put differently, if a given unicast packet can only be sent on Tx supercells tagged with a certain TSCH mode, other Tx supercells to the same neighbor cannot be used for the transmission of other unicast packets as long as we are attempting to transmit the current packet. As such, if we allowed non-probe unicast packets on negotiated Tx supercells only, retransmissions of those packets would often cause us to skip the Tx supercells tagged with alternating mode, thereby preventing us from maintaining fresh inferred metrics.

If the Tx supercells tagged with the alternating TSCH mode (i.e., to be used towards neighbors we already have negotiated Tx supercells towards) were autonomous Tx supercells, they would be shared. Since non-probe unicast transmissions would be allowed on these supercells, this would be especially problematic in networks with a tree-like routing topology and mostly MP2P traffic. Hence, we required another slotframe based on the alternating TSCH mode: the probing slotframe. The probing slotframe is managed in a similar fashion to the negotiated slotframe (see Section 4.5). As with negotiated supercells, probing supercells also correspond to dedicated links. Probing supercells can only overlap with autonomous Tx supercells, and the probing slotframe handle must be greater than the autonomous slotframe handle but less than the negotiated slotframe handle.

A node must install exactly one probing Rx and one probing Tx supercell towards a newly preferred parent (in that order) before it can install a negotiated Tx supercell towards the same parent, and both probing supercells must remain scheduled as long as at least one negotiated Tx supercell is scheduled towards the same neighbor. Additionally, a node cannot have an autonomous Tx supercell towards neighbors it already has a probing Tx supercell towards. All unicast traffic is allowed on both probing and negotiated supercells. That said, probes may be dropped in favor of other unicast packets under certain conditions (see Section 4.6).

MSF’s windowed counter mechanism for negotiated cell management [7] (Section 5.1) is problematic for negotiated Rx cells because the owner of an autonomous Rx cell does not know when the owner of the corresponding autonomous Tx cell backs off (see Appendix B). Therefore, 3MSF updates this mechanism slightly. More specifically, when there is no negotiated Rx supercell towards the preferred parent, the probing Rx supercell (as well as the packets received on it) and not the autonomous Rx supercell (nor the packets received on it from the preferred parent) is taken into account for downstream traffic adaptation. This works because the probing Rx supercell towards the preferred parent is mandatory (so we can maintain fresh inferred metrics towards the preferred parent) but (unlike autonomous supercells) the corresponding probing Tx supercell (as seen from the preferred parent’s perspective) is not shared.

Finally, this brings us to the shadow slotframe, which is more of a pseudo-slotframe, as shadow supercells are not considered when determining the next link to use (hence the slotframe handle *∞*). As far as the processes that govern the selection of the next link are concerned (see Section 4.4), the shadow slotframe does not exist. The sole purpose of the shadow slotframe is to build (in parallel) an alternative to the current negotiated slotframe towards the preferred parent if we wish to change the interface-mode to be used in the negotiated cells towards it. Shadow supercells can only overlap with autonomous Tx supercells and negotiated supercells, regardless of type.

The sequence of 6P transactions required to switch the interface-mode used in the negotiated supercells towards the preferred parent is called the “shadow operations”. Section 4.5 explains which transactions make up the shadow operations, and Section 4.6 explains when shadow operations with the preferred parent are triggered.

### 4.3. Timeslot Timings and Supercell Length

3MSF uses variable-duration timeslots by aggregating consecutive “unit-duration” subslots in a superslot. The timing intervals to use within a superslot are (mostly) dictated by the data rate of the communication mode(s) it uses and the maximum packet size for said communication mode(s). After all, by definition, each (super)slot must allow for the exchange of a unicast packet and a corresponding acknowledgement (ACK).

**Figure 6:**
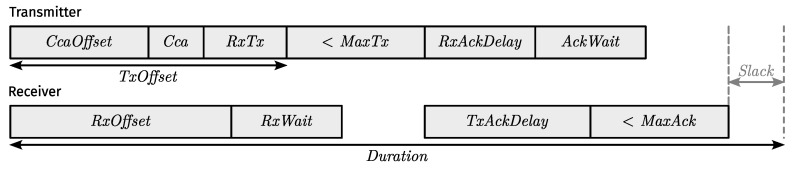
Timeslot timing intervals assuming acknowledged transmission. A unique set of timing intervals is associated with each supercell length. Any interface-mode that wishes to use supercells of a given length must be able to transmit a unicast packet and receive a corresponding acknowledgement using the corresponding timing intervals.

Figure 6 shows the layout of TSCH timing intervals for acknowledged transmission [2] (Section 6.5.4.2). Knowing which values to use starts with determining appropriate lower/upper limits for the timing intervals of each non-simultaneous TSCH mode. It is important to remember that a non-simultaneous TSCH mode maps to a single communication mode, as explained in Section 4.1.

All relationships between the different timing intervals are listed in (4) or can otherwise be derived from it.

(4) $$\left\{\begin{array}{c} RxTx>RxWait/2\\ MaxTx>RxWait/2\\ RxOffset=TxOffset-RxWait/2\\ TxOffset=CcaOffset+Cca+RxTx\\ RxAckDelay=TxAckDelay-AckWait/2\\ TxAckDelay>AckWait/2\\ MaxAck>AckWait/2\\ Duration=TxOffset+MaxTx+TxAckDelay+MaxAck+Slack \end{array}\right.$$

where:
RxTxthe time between performing (optional) Clear Channel Assessment (CCA) and the start of transmission;RxWaitthe receiver guard time;MaxTxthe time needed to transmit packet of maximum size;RxOffsetthe time to the earliest possible start of reception;TxOffsetthe time to the start of transmission;CcaOffsetthe time to the start of (optional) CCA by the transmitter;Ccathe duration of (optional) CCA by the transmitter;RxAckDelaythe smallest possible time between the end of transmission and the start of ACK reception;TxAckDelaythe time between the end of reception and the start of ACK transmission;AckWaitthe transmitter guard time (not used for synchronization);MaxAckthe time needed to transmit an ACK of maximum size;Slackmust be greater than or equal to the time needed for the remaining operations and going to sleep.

Once you have determined appropriate ranges for the timing intervals of each non-simultaneous TSCH mode, you must decide on the exact intervals that make up a unit-length superslot and together amount to unit duration. The supercell length used for a given non-simultaneous TSCH mode determines how to derive the timing intervals from the intervals of a unit-length superslot. While superslot duration is an integer multiple of unit duration, where the multiple is the superslot/supercell length, the timings that make up superslot duration are not necessarily multiplied by the supercell length. That is, we require CCA, RxTx, RxWait, and AckWait to remain constant. In addition, TxOffset and TxAckDelay each differ from their unit-duration counterparts by a unique factor. Similarly, MaxTx and MaxAck differ from their unit-duration counterparts by the same factor. Afterwards, the remaining values can be determined using (4).

Determining appropriate supercell timings and lengths is an exercise in scheduling efficiency, as the best choice largely depends on the spread of data rates across communication modes. It should be noted that all nodes must have a common understanding of the supercell length to use for each non-simultaneous TSCH mode (which themselves map to a single communication mode), either by statically configuring them with such information a priori or through some mechanism that is beyond the scope of this paper.

**Figure 7:**
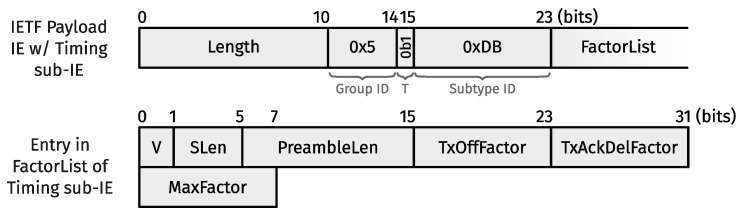
Format of a TSCH IETF Informational Element (IE) carrying a Timing sub-IE. Note that we used an IETF IE Subtype ID [12] (Section 4) in the experimental usage range. The Timing sub-IE contains a variable-length FactorList, where each entry must follow a specific format.

Unit-length superslot timings are disseminated conventionally, i.e., with the TSCH Timeslot IE [2] (Section 7.4.4.4) in EBs. Similarly, we designed a new sub-IE for the IETF IE, which we termed the “Timing” sub-IE, to carry the three coefficients used to derive TxOffset, TxAckDelay, MaxTx, and MaxAck from their unit-duration counterparts. More specifically, as shown in Figure 7, the Timing sub-IE contains a variable-length FactorList, where each entry follows a specific format. That is, each entry maps a supercell length greater than 1 to three 8-bit fixed-point scaling factors. The two factors for TxOffset and TxAckDelay have six spaces behind the binary point, whereas the scaling factor for MaxTx and MaxAck has four. Support for supercell lengths greater than 1 requires an IETF IE with a Timing sub-IE in every EB.

In Section 4.1, we mentioned how the supercell length for a simultaneous TSCH mode equates to the longest supercell length we would ordinarily use for any non-simultaneous TSCH mode corresponding to the communication modes to which it (i.e., the simultaneous TSCH mode) points. As always, its length dictates the timing intervals to use in a supercell. As such, for simultaneous transmission, the same timings apply to all underlying communication modes, which makes synchronization relatively straightforward. That is, assuming transmission is truly simultaneous at the link layer, we need only derive the drift from the first ACK (with ACK-based synchronization) or the first packet (with frame-based synchronization) received.

### 4.4. TSCH Link Selection

**Figure 8:**
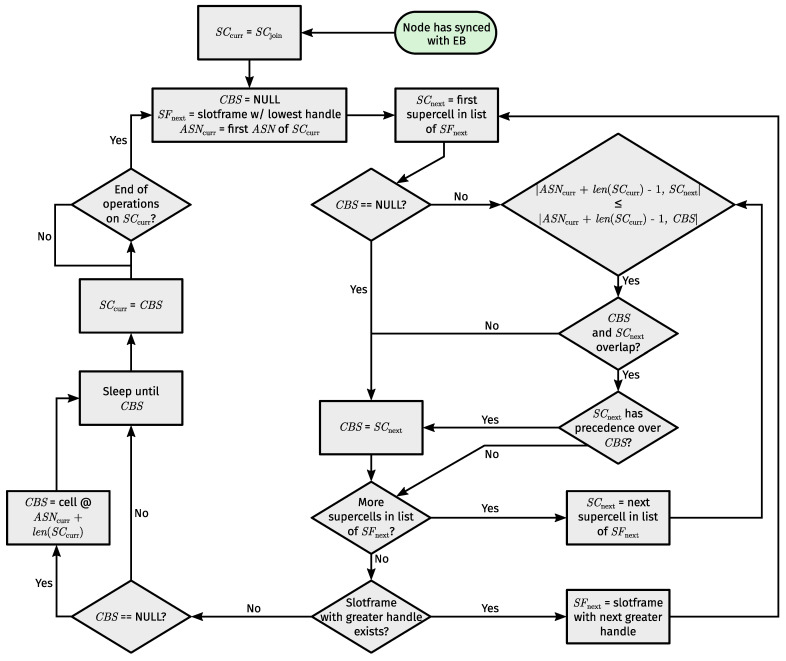
Flowchart of events related to the selection of the next link a node must wake up for when it has either just joined the TSCH network by synchronizing to an Enhanced Beacon (EB) or when operations on the currently active link have concluded. In this flowchart, $SC_{curr}$ is the currently active supercell, $SC_{join}$ is the minimal supercell in which the node first synchronized to an EB and joined the TSCH network, $SC_{next}$ is the supercell being evaluated against the Current Best Supercell (CBS) during link selection, $SF_{next}$ is the slotframe to which $SC_{next}$ belongs, and $ASN_{curr}$ is the Absolute Slot Number (ASN) of $SC_{curr}$’s first subcell.

Supercells require a slightly modified TSCH link selection process. Presumably, the next active link (and hence how long a node can sleep) is determined when operations on the current link have concluded. At that point, a node iterates through all slotframes in ascending order of slotframe handles. For each supercell encountered, the node calculates how many slot offsets its first subcell is removed from the last subcell of the current supercell. At the same time, the node tracks a single supercell, which we call the Current Best Supercell (CBS). That is, the first supercell the node encounters automatically becomes the CBS. Then, for every next supercell, it compares the distance to the CBS with the distance to the given supercell. If the distance to the given supercell is less than or equal to the distance to the CBS and there is no overlap between them or the given supercell has precedence (see Section 2.1.2), the given supercell becomes the CBS. Finally, the node is left with the most appropriate next link to wake up for. Figure 8 puts the process of next active link selection in a handy flowchart.

A backup link may also be selected when retrieving the next active link. Normally (i.e., without supercells) a backup link is selected from all Rx links that coincide with the ultimately selected next active link. Selection happens according to the lowest slotframe handle among all eligible backup links (since only Rx links are eligible). However, when using supercells, only Rx supercells that start at the same slot offset as the ultimately selected next active supercell, and fall entirely within said supercell, are eligible as a backup supercell.

### 4.5. 6P Transactions

3MSF uses the Metadata field of 6P request messages [11] (Section 3.3) to indicate to which slotframe a request pertains. For the probing and shadow slotframe, the Metadata field value equals the respective slotframe handle, whereas for the negotiated slotframe, the value is zero. In Section 4.2, we mentioned that the shadow slotframe is effectively a pseudo-slotframe with a handle of *∞*. In practice, you would simply give the shadow slotframe a handle (e.g., 5) and treat it as an escape value.

**Figure 9:**
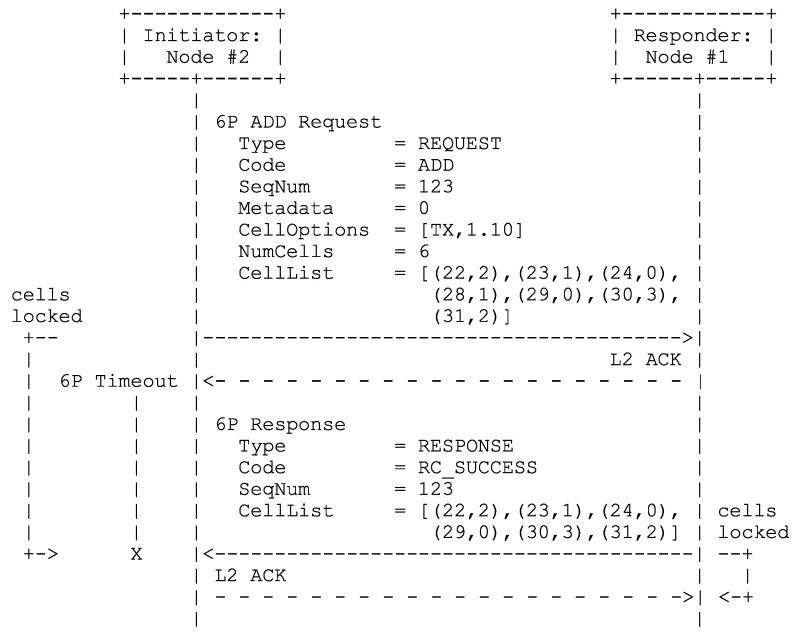
Example of a successful 6P ADD transaction used by 3MSF, based on the fictitious setup depicted in Figure 5. Node #2 sends an ADD request to its preferred parent #1, providing it with a CellList containing two subcell groupings. From these groupings, node #1 picks two supercells in the negotiated slotframe following the request’s NumCells value and the supercell length corresponding to the TSCH mode specified by node #2 in the 3 MSBs of the CellOptions.

Figure 9 shows a possible ADD transaction, which works the same for the probing, negotiated, and shadow slotframes. Node #2 wants to install two negotiated Tx supercells towards its preferred parent #1, forms an ADD request with a Metadata field value of 0, and populates its CellList [11] (Section 3.2.4) with groupings of consecutive subcells. Each subcell grouping must accommodate at least one supercell of the length required by the TSCH mode contained in the three MSBs of the request’s CellOptions field. The NumCells field indicates how many subcells from the CellList #2 wishes to install towards #1 in the slotframe pointed to by the Metadata field. Hence, NumCells must be an integer multiple of the required supercell length, and the subcell groupings in the CellList must accommodate at least this integer multiple of supercells. Upon receiving the ADD request and performing all the checks required by 6P, #1 verifies that (1) NumCells is an integer multiple of the required supercell length; (2) each subcell grouping has a length greater than or equal to the supercell length; and (3) the proposed subcell groupings can accommodate a number of supercells greater than or equal to NumCells divided by the required supercell length. If the additional verification fails, or if #1 has no space left in its schedule to pick at least one supercell from the subcell groupings proposed by #2, #1 returns a 6P response containing the code RC_SUCCESS and an empty CellList. Otherwise, #1 returns an RC_SUCCESS response with a non-empty CellList, which is less than or equal to NumCells in length, and where all groupings are integer multiples of the required supercell length. It should be noted that it seems more appropriate to return a 6P response containing the code RC_ERR_CELLLIST if the additional checks were to fail. Unfortunately, this would violate RFC 8480 [11] (Section 3.3.1).

Very similar to ADD transactions are DELETE transactions, which 3MSF only uses for the negotiated slotframe. We retain all rules from RFC 8480 [11] (Section 3.3.2) concerning the deletion of cells (using a two-step 6P transaction). Additionally, we require (1) all subcell groupings to match actual supercells; and (2) NumCells to be an integer multiple of the required supercell length.

**Figure 10:**
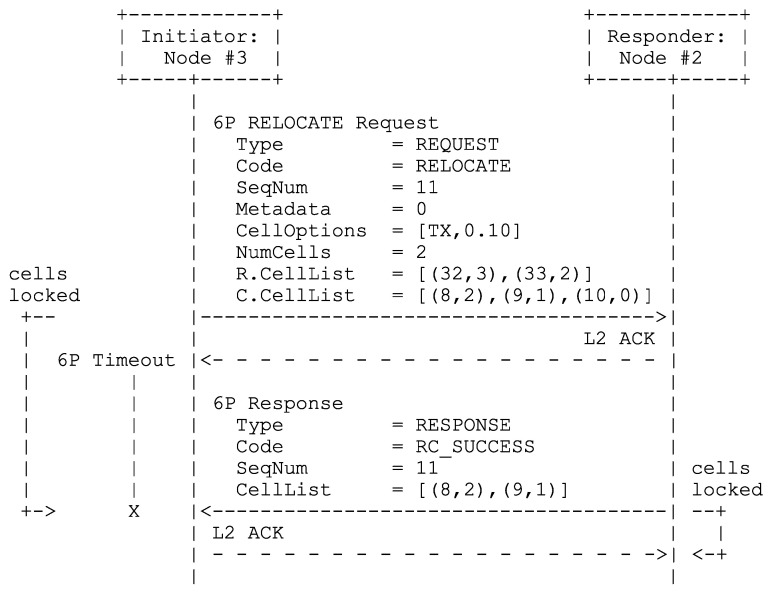
Example of a successful 6P RELOCATE transaction used by 3MSF, based on the fictitious setup depicted in Figure 5.

3MSF also supports 6P RELOCATE transactions for the relocation of negotiated and probing supercells. We retain all rules from RFC 8480 [11] (Section 3.3.3) concerning the relocation of cells (using a two-step 6P transaction). Additionally, we require (1) all subcell groupings in a RELOCATE request’s relocation CellList, as well as those in the response’s CellList (if non-empty), to match actual supercells; (2) NumCells to be an integer multiple of the required supercell length; and (3) the subcell groupings in a RELOCATE request’s candidate CellList to each have a length greater than or equal to the supercell length and together be able to accommodate greater than or equal to the integer multiple of supercells specified in (2). Figure 10 shows an example of a possible (successful) RELOCATE transaction for 3MSF.

CLEAR transactions work nearly identically between 3MSF and MSF, with one small caveat. A CLEAR request with a Metadata field value of zero removes all supercells (towards the same neighbor) from the negotiated, probing, and shadow slotframes. Such a “general” CLEAR request/transaction is used if there is any risk of an inconsistent negotiated slotframe. Otherwise, a slotframe-specific CLEAR request is used.

Finally, this leaves us with the sequence of 6P transactions we call shadow operations, which can only be initiated by a node towards its preferred parent under the following conditions:

1. Shadow operations initiated by said node are not already ongoing (previous operations need to be terminated first; further details on this are discussed later);
2. A non-shadow 6P transaction is not currently ongoing with the preferred parent in any direction;
3. A non-shadow 6P transaction is not already scheduled to be initiated at some point in the future (e.g., because of a retry);
4. There is not already a maximum number of concurrent 6P transactions ongoing;
5. The node has a probing Tx supercell towards its preferred parent.

**Figure 11:**
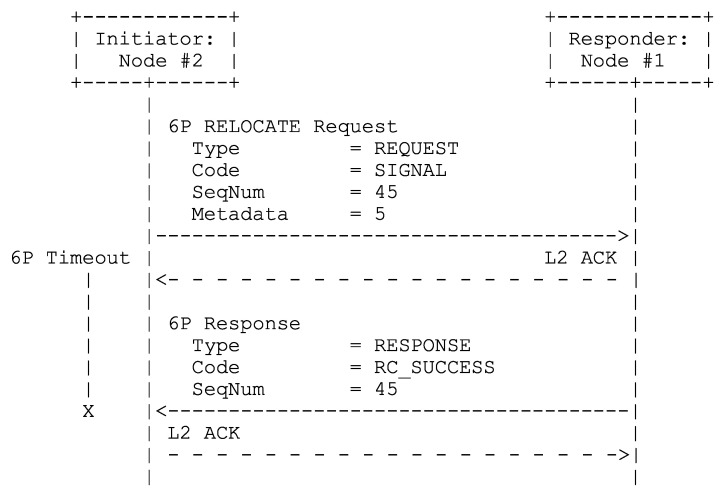
Example of a successful 6P SIGNAL transaction used by 3MSF during shadow operations, based on the fictitious setup depicted in Figure 5. Node #2 sends a SIGNAL request to its preferred parent #1, indicating that the supercells in the shadow slotframe between #2 and #1 (previously added through shadow ADD transactions) are ready to replace the existing negotiated supercells between #2 and #1. The successful completion of this SIGNAL transaction marks the end of shadow operations.

During shadow operations, a node first determines how many negotiated Tx supercells it has towards its preferred parent before sending it an ADD request for (at most) the same number of shadow Tx supercells. Upon successfully installing at least one shadow Tx supercell (a maximum number of retries is allowed until at least one such supercell is added), the node may do the same for negotiated Rx supercells (if there are any, the same rules apply for retries). Finally, the node initiates a custom two-step SIGNAL transaction (e.g., see Figure 11). When this transaction completes successfully (with the same rules applying for retries) and not at any other point in time, all negotiated supercells involved are removed, new negotiated supercells are derived from the shadow supercells towards said neighbor, those shadow supercells are removed, and shadow operations terminate (in that order).

Since the interface-mode used in negotiated supercells performed poorly, all traffic belonging to shadow operations must be sent using probing supercells only. Unfortunately, this creates a minor bottleneck due to the MAC sequence number consistency requirements mentioned in Section 4.2.

During shadow operations, the following rules apply (besides any rules mentioned previously):

1. The windowed counter mechanism for negotiated supercell management (see Section 4.2) is halted, and its counters are reset;
2. No non-shadow 6P transactions towards the preferred parent (with the exception of CLEAR transactions under appropriate circumstances) may be initiated towards the preferred parent or be scheduled for future initiation.

Switching the frozen interface-mode to the preferred parent (which triggers new shadow operations, see Section 4.6) or switching the preferred parent while shadow operations with it are ongoing causes them to terminate, i.e., by aborting any ongoing non-CLEAR transactions belonging to those shadow operations and initiating appropriate CLEAR transactions. Receiving a response code requiring a shadow transaction be aborted and a CLEAR request (of any kind) be sent to the preferred parent (e.g., because it detected an inconsistency), or exceeding the retry limit for a non-CLEAR shadow request, has a similar effect.

### 4.6. Related to Metrics

As explained in Section 2, supporting multi-modal operation affects all layers that derive functionality from link-layer operation. With 6TiSCH, the chosen RPL OF imposes requirements on the TSCH SF, and vice versa. Based on the premise of avoiding excessive parent changes/local repairs [1], we combined 3MSF with a slightly adapted version of our Dual Radio-Interface Routing Protocol for LLNs (DRiPL) OF, referred to as DRiPLOF [8].

**Figure 12:**
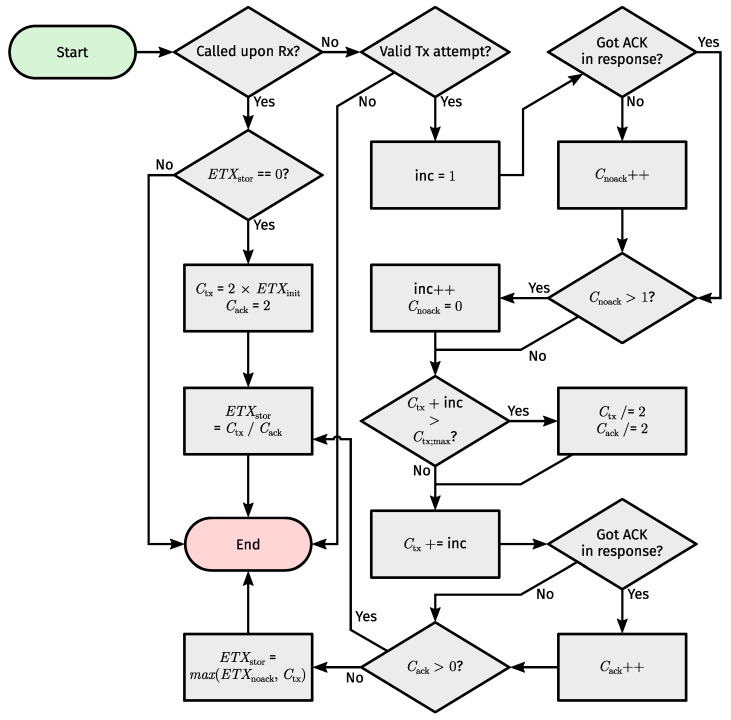
Flowchart of events when the inferred metric update process is called for a given interface-mode of a given neighbor. $C_{tx}$ and $C_{ack}$ are the transmit (Tx) and acknowledgement (ACK) counters, $C_{tx;max}$ is the Tx counter’s maximum value, $C_{noack}$ counts consecutive non-ACKed Tx attempts, $ETX_{init}$ is a constant used to initialize the inferred metric when receiving on a newly discovered interface-mode, $ETX_{stor}$ is the actual inferred metric, and $ETX_{noack}$ is a large constant, which must be greater than the inferred metric threshold. A separate set of these variables is maintained for each interface-mode of each neighbor.

Most changes applied to DRiPLOF relate to routing metrics (see Appendix C for other differences). Physical links [8] (Section 4.2) (which are distinct from TSCH links) now equate to interface-modes, and inferred metrics now belong to interface-modes. We use the Expected number of Transmissions (ETX) [10] (Section 4.3.2) as the inferred metric. This means that we still require a metric threshold to asses an interface-mode’s status. However, as different Tx attempts of the same packet can now occur on different interface-modes, we can no longer just update ETX when a (unicast) packet is dequeued. Instead, we perpetually calculate ETX for each interface-mode as the ratio of a Tx and ACK counter, both of which are tied to a single interface-mode of a given neighbor and are updated after each Tx attempt across said interface-mode. In addition, we penalize ETX if we fail to transmit on an interface-mode for a number of consecutive attempts, regardless of attempts on other interface-modes. To qualify as a Tx attempt, a packet must have been on-air or its transmission must have been postponed because CCA failed (CCA is not mandatory). We consider CCA failures as Tx attempts to mitigate denial-of-service attacks through jamming, as only Tx attempts lead to inferred metric updates. Figure 12 illustrates the flow of events when the process governing inferred metric updates is called.

When an inferred metric changes, the preferred interface-mode towards the corresponding neighbor is (re-)selected. However, since the interface-mode used for unicast transmissions is now dictated by the supercells they occur in, the preferred interface-mode is no longer automatically used for all unicast transmissions towards a given neighbor. That is, swapping out the interface-mode to use in negotiated supercells towards the preferred parent on every preferred interface-mode change is not feasible. Thus, performing such a switch is rate-limited. We call the interface-mode to use in all negotiated supercells towards the preferred parent the “frozen” interface-mode.

A frozen interface-mode switch can only occur when the inferred metric of the currently preferred and/or frozen interface-mode towards the preferred parent changes. If a node does not have any negotiated supercells towards its preferred parent and it has not yet initiated a (currently ongoing) 6P transaction to add such supercells, shadow operations are not needed, and the node can simply alter the CellOptions field of the 6P ADD request it will supposedly send to its preferred parent soon. In this case, a node must switch frozen interface-mode if the inferred metric of the currently preferred interface-mode towards the preferred parent is better than the inferred metric of its frozen interface-mode, i.e., without applying hysteresis. Otherwise, shadow operations are always triggered when a frozen interface-mode switch is required for one of the following reasons:

- If the frozen interface-mode went down, switch to the currently preferred interface-mode if that one is up.
- If neither the frozen nor the currently preferred interface-mode is down but the absolute difference between their inferred metrics exceeds a hysteresis threshold, switch to the preferred interface-mode.

If the preferred interface-mode towards the preferred parent fails, a preferred parent switch will occur soon and shadow operations cannot be triggered.

Finally, the path cost through a candidate neighbor is the sum of the normalized metric towards it and (because we use ETX) the rank it advertises. Metric normalization consists of (1) calculating a weight based on the number of unavailable interface-modes towards a neighbor using (5), and (2) calculating the normalized metric using (6).

(5) $$W=\left\{\begin{array}{cc} 0 & \Leftrightarrow min\left(IM_{node}-VL,IL_{max}\right)=0\\ \frac{min\left(IM_{node}\;-\;VL,\;IL_{max}\right)}{IL_{div}}-W_{off} & \Leftrightarrow min\left(IM_{node}-VL,IL_{max}\right)>0 \end{array}\right.$$

(6) $$M_{norm}=W\times S+\left(1-W\right)\times M_{inf}\;,$$

where:
*W*the metric normalization weight;$IM_{node}$the number of interface-modes per node;VLthe number of valid physical links towards a given neighbor;$IL_{max}$the maximum number of invalid physical links towards any neighbor, must be ≥0 and $<IM_{node}$;$IL_{div}$a constant divider, which must be $>IL_{max}$;$W_{off}$a weight offset, which must be ≥0 and $\leq 1/IL_{div}$;$M_{norm}$the normalized metric towards a given neighbor;*S*a constant scale multiplier;$M_{inf}$the inferred metric of either the frozen interface-mode (to the preferred parent), or the preferred interface-mode (to other neighbors).

The way we calculate weights using (5) is slightly modified from [8] (Equation (2)). That is, we introduce a weight offset $W_{off}$ term to allow for additional fine-tuning.

Enough unicast packets must be sent to all neighbors on all interface-modes such that their normalized metric (and therefore, path cost) is always a reasonably accurate reflection of their capabilities. Since an interface-mode’s inferred metric is updated after each unicast Tx attempt on a supercell tagged with a TSCH mode corresponding to (at least) said interface-mode, and there is often a non-equal amount of Tx opportunities per interface-mode towards the same neighbor over time, maintaining fresh inferred metrics without incurring excessive overhead is tricky, especially considering the MAC sequence number consistency requirements discussed in Section 4.2.

The mechanism governing reachability detection/metric freshness generally determines a target (i.e., a neighbor). In our case, the same neighbor must remain the target until we have reached a minimal number of Tx attempts on all its interface-modes. Although a Tx attempt of any unicast packet will update an inferred metric, some unicast packets only serve to determine reachability through an interface-mode and/or to maintain a fresh inferred metric for it. Sending such packets over interface-modes with already up-to-date inferred metrics is wasteful.

Contiki-NG [18], the Operating System (OS) we based our firmware on when designing and evaluating our 6TiSCH architecture, normally (i.e., in single-mode configuration) uses unicast RPL control messages as probes (we used unicast DODAG Information Solicitation (DIS) messages because DIOs advertise DODAG connectivity, which is not allowed by 3MSF prior to said node having at least one negotiated Tx (super)cell towards its preferred parent) to determine neighbor reachability and obtain metrics, as opposed to IPv6 Neighbor Unreachability Detection (NUD) [19] (Section 5.5). However, the probing of a given neighbor completes as soon as a unicast packet towards it (probe or not) is dequeued following a valid Tx attempt. As such, the probing mechanism was modified. Although the principles behind sending enough packets on each interface-mode of a neighbor are likely very similar regardless of the mechanism, we limit our discussion to Contiki-NG probing.

**Figure 13:**
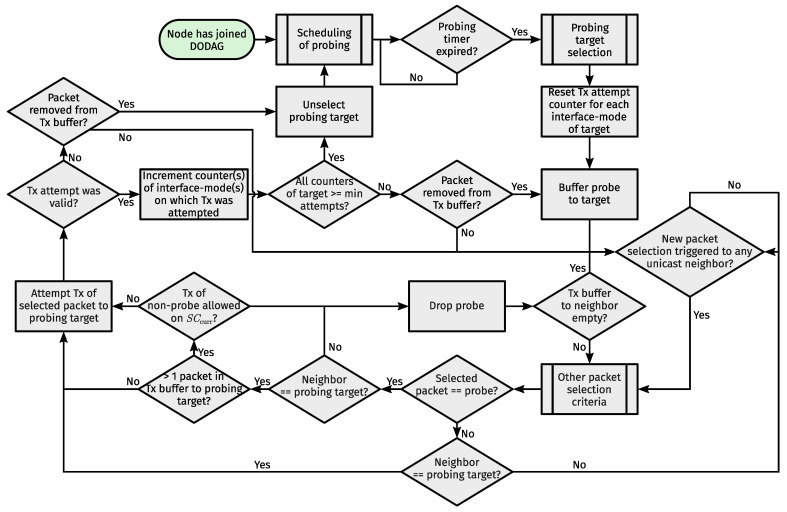
Updated flowchart of events involved in Contiki-NG probing. The idea is to perform a minimal number of Tx attempts on each interface-mode of a probing target before the probing of said target completes, such that we can reasonably assume all its inferred metrics are fresh upon completion while incurring a minimal amount of overhead.

Figure 13 illustrates the updated flow of events involved in Contiki-NG probing. Upon selecting a new probing target, a unicast probing packet is buffered towards it. For every interface-mode of the target, we count the number of unicast Tx attempts since its selection. When the required number of Tx attempts is reached for every interface-mode of the current probing target, it is unselected. When a packet for the current probing target is removed from the corresponding Tx buffer upon a valid Tx attempt, a unicast probe is added to that buffer. When a unicast packet towards a neighbor is retrieved from the corresponding Tx buffer, the unicast packet that would otherwise (i.e., through other criteria) be sent next is first inspected. That is, if it is a probe but the neighbor is no longer a probing target, or there is more than one packet in the buffer and the supercell to send it on is eligible for transmission of non-probe unicast packets, the probe is dropped, and another unicast packet is retrieved (which is then also inspected). This way, we do not violate the MAC sequence number consistency requirements or create excessive overhead.

## 5. Simulations and Results

As a basic proof of concept for 3MSF (see Section 4), we devised three simple scenarios, which we then evaluated through a combination of heavily modified Contiki-NG firmware and the Cooja simulator [20]. All our modifications of/additions to Cooja and Contiki-NG are open source, allowing anyone to use our code for future multi-modal 6TiSCH research. More information is available in the Supplementary Materials and the appropriate branches of our GitHub forks of Cooja [21] and Contiki-NG [22], respectively.

Concerning Cooja, we built upon our prior adaptation for multi-interface support to now also support multiple communication modes per interface, as well as simultaneous transmission towards the same neighbor (on at most one mode per interface, interfaces are assumed to operate in different frequency bands). As in our prior work [8] (Section 5.2), we used a log-distance path loss model to simulate the electromagnetic properties of the medium. Table 3 lists all the constants we used to configure said model.

We also added the capability to “break” specific physical links between a given pair of nodes for a given amount of time and in a given direction, meaning that all packets sent on said physical link (regardless of channel) and in said direction will not arrive during said time period. The purpose of doing so depends on the scenario.

Regardless of the scenario, each node was configured with the same four interface-modes listed in Table 3, unless noted otherwise. As explained in Section 4.3, each interface-mode maps to a non-simultaneous TSCH mode, which, in turn, map to supercell lengths, each of which corresponds to a set of three coefficients used for deriving TxOffset, TxAckDelay, MaxTx, and MaxAck from their unit-duration counterparts. Table 4 lists the mappings used, whereas Table 5 lists the timing interval values of a unit-duration timeslot.

As long as a non-root node is part of the DODAG and has at least one negotiated Tx supercell towards its preferred parent, it (pseudo-)periodically sends data packets to the root. That is, a primary timer expires every 12 s, and upon expiration, a secondary timer is set to a random time less than or equal to 6 s past the primary timer’s last expiration. When the secondary timer expires, a node determines whether it can send a data packet to the root. The root is configured not to send responses to these data packets downstream.

Table 6 lists the most important configuration parameters other than the timeslot timings/supercell lengths common to all scenarios unless noted otherwise.

### 5.1. Scenario 1: Two Nodes

**Figure 14:**
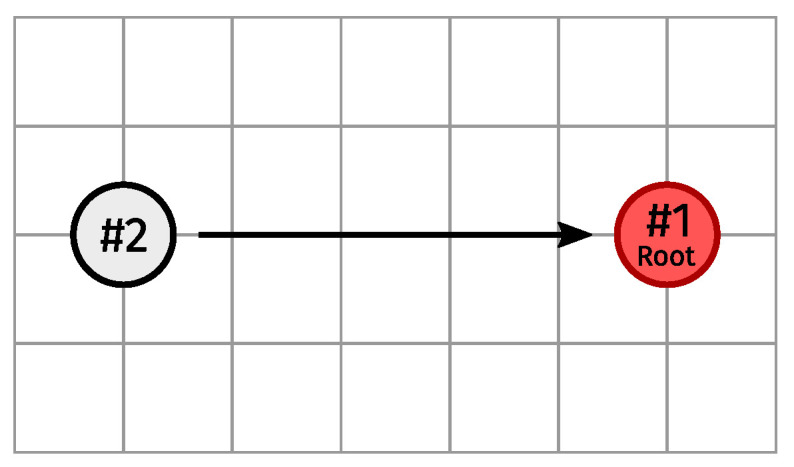
Setup of the first scenario involving two nodes, i.e., a root (#1) and a non-root node (#2). The black arrow indicates a child–parent relationship. A single grid square measures 10 m × 10 m.

Figure 14 illustrates the first scenario, which consisted of two nodes, a root (#1) and a non-root node (#2), separated by 50 m. The network operated under normal conditions for 10 min to reach a steady state. Then, from the 10 min mark, we blocked all sub-GHz communication modes between nodes #1 and #2 in both directions in order to emulate adversities impacting all interface-modes that operated in the same frequency range (on all channels of that range), such as wide-band interference/jamming.

**Figure 15:**
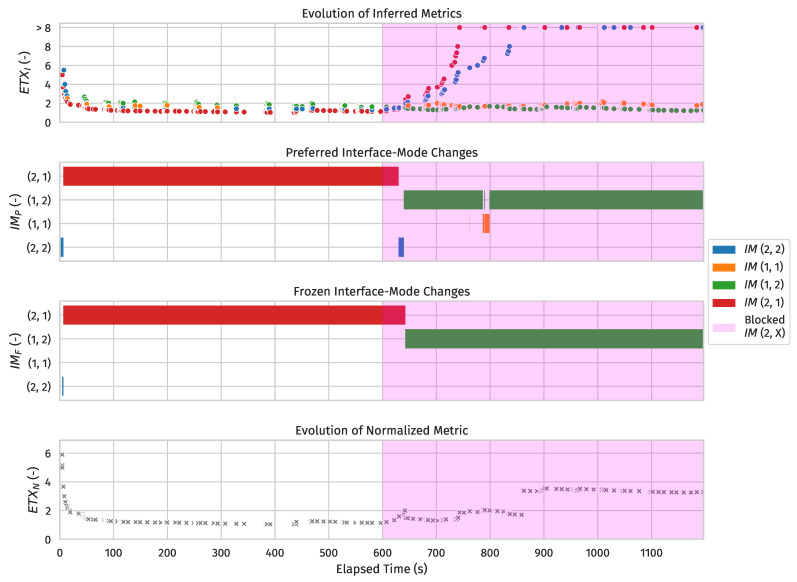
Characterization of the “link” between nodes #2 and #1 (i.e., the root), as depicted in Figure 14, for a single simulation run. The first graph shows the evolution of the inferred metrics for each interface-mode over time. The second graph depicts the time intervals in which a given interface-mode was preferred. The third graph depicts the same but for the frozen interface-mode. The fourth and final graph shows the evolution of the normalized metric over time. In each graph, the pink highlighted area shows the time period during which all sub-GHz interface-modes were blocked.

Figure 15 characterizes the (virtual) link between nodes #2 and #1 based on the data gathered from a single simulation run and only serves to illustrate several key mechanisms of our 6TiSCH implementation in action. As can be observed, the first normalized metric for #1 was equal to $ETX_{init}$, meaning #2 received a packet (most likely an EB) on all interface-modes it shared with #1 (which was all of them) prior to transmitting a unicast packet to #1 on any of them.

The relatively high $ETX_{init}$ value was required because we found that the odds of successfully transmitting a unicast packet on (shared) autonomous Tx supercells were generally lower than the odds of doing so on (dedicated) probing/negotiated Tx supercells. As such, the high $ETX_{init}$ value prevents a newly discovered node and/or a node with newly discovered interface-modes from becoming the preferred parent only to immediately have its inferred metrics (and by extension, normalized metric and path cost) increase significantly upon initiating a 6P ADD transaction for the probing Tx supercell towards it (the traffic of which is all sent on autonomous supercells), which would lead to instability. However, it is important, especially during the network startup phase, that the ETX of all frozen interface-modes along the path to the root decreases quickly enough (from $ETX_{init}$, where applicable). Otherwise, newly discovered nodes with worse virtual links but shorter paths to the root than the theoretically ideal preferred parent (i.e., in steady state) would wreak havoc on network convergence speed.

The normalized metric bump around the 640 s mark shows the frozen interface-mode switching mechanism in action. At that point, all inferred metrics to #1 were below the metric threshold, so #1’s normalized metric equated to its frozen interface-mode’s inferred metric. The bump was hence caused by said inferred metric rising until the inferred metric difference with #1’s preferred interface-mode exceeded FROZEN_SWITCH_THRESHOLD. It is worth noting that the frozen interface-mode may seem unstable at the beginning. However, it is important to remember that a node can switch the frozen interface-mode towards its preferred parent at will when it has no negotiated Tx supercell towards it or when a 6P transaction to add such a supercell(s) is not ongoing.

Finally, each time another sub-GHz interface-mode exceeded the inferred metric threshold, the normalized metric was clearly penalized for said interface-mode going down following (5) and (6). That is, while the inferred metric of the frozen interface-mode remained relatively constant, the penalty applied to #1’s normalized metric was visibly proportionate to the number of unavailable interface-modes.

**Figure 16:**
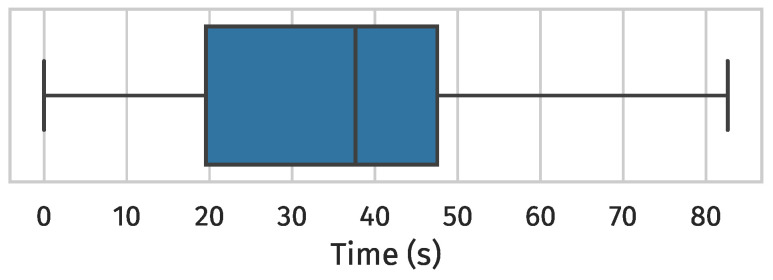
Time to non-blocked interface-mode from the start of blocking all modes of the 868 megahertz (MHz) interface at once (i.e., interface two) across all simulations in this scenario. The rare cases where a 2.4 GHz interface-mode becomes frozen first but the absolute difference in inferred metric never exceeds the hysteresis threshold explain the 0 values.

Figure 16 shows the time it took for node #2 to switch from its frozen interface-mode towards #1 at the 10 min mark (i.e., when we broke all sub-GHz physical links) to any interface-mode in the 2.4 GHz band across all simulations. In 37 out of 40 simulations, #2’s frozen interface-mode towards #1 at the 10 min mark was sub-GHz, as the opposite can only happen if a 2.4 GHz interface-mode becomes frozen first and the absolute difference in inferred metric never exceeds the hysteresis threshold, which is rare. Nonetheless, this did happen, which explains the 0 values. It is important to note that the average time to a frozen interface-mode switch is not particularly fast, but this is mainly governed by the hysteresis threshold. You do not want to switch too fast to prevent excessive overhead (from shadow operations).

### 5.2. Scenario 2: Three Nodes

**Figure 17:**
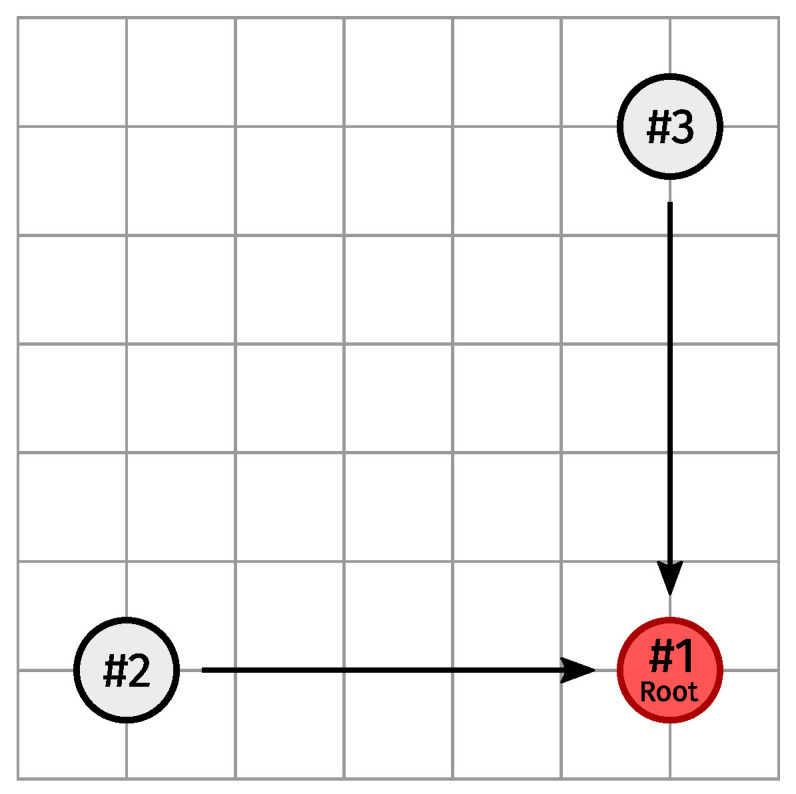
Setup of the second scenario involving three nodes, i.e., one root (#1) and two non-root nodes (#2, and #3). The black arrows indicate (the intended) child–parent relationships. A single grid square measures 10 m × 10 m.

Figure 17 depicts the setup of the second scenario, in which two non-root nodes (#2, and #3) were positioned 50 m away from the root node (#1) along the x- and y-axes, respectively (meaning #2 and #3 were separated by ≈70.7 m). This scenario did not involve breaking any physical links. Instead, the network was simply left to operate under normal conditions during the entire 20 min simulation run (we ran 40 simulations of this scenario in total).

**Figure 18:**
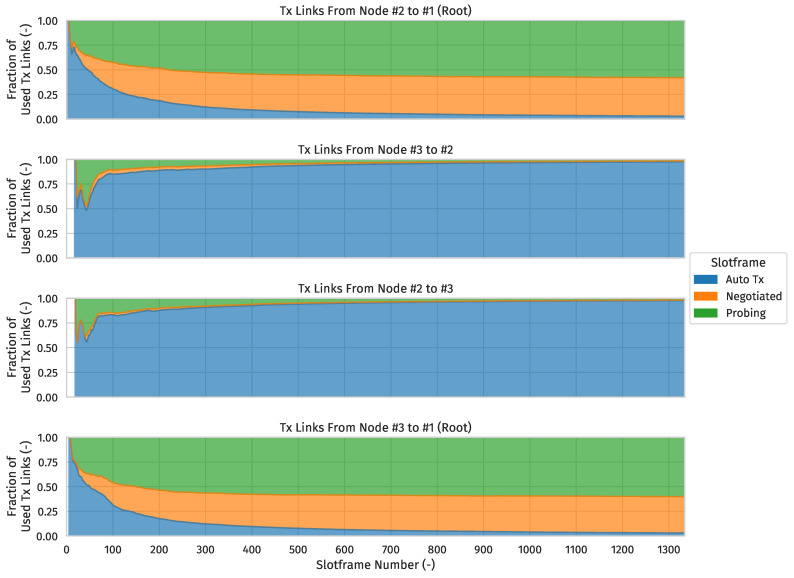
Cumulative number of used transmit links across all simulations of this scenario (represented as a fraction) by slotframe. Only Tx links of directed node pairs in which the destination could potentially be the preferred parent of the source are displayed.

For each slotframe number, Figure 18 gives an impression of the fraction, by slotframe handle, of the total number of used Tx links since the start of the network from the root’s perspective (assuming the slotframe in which the root broadcasts its first EB is the slotframe with number 0, and so on). Put differently, the slotframe number indicates how many 3MSF slotframes have elapsed since the root started advertising, and for any such point on the x-axis, the y-axis is divided according to the fraction of the total number of used Tx links (from a single given node to a single given neighbor) up to and including that point which were of a certain type.

As you can see from the cumulative negotiated Tx link usage from #2 to #3, and vice versa, evolving to 0 (and being very small to begin with), the intended child-parent relationships were eventually achieved in all cases (and immediately in most).

Additionally, the graphs show the probing Tx supercell to the preferred parent being used slightly more often than the negotiated Tx supercell(s). That is, due to the relatively low amount of upstream traffic in this very small network, there was only one negotiated Tx supercell to the preferred parent, and the probing mechanism comparatively needing more Tx opportunities on probing supercells to obtain fresh metrics for all interface-modes of the preferred parent hence had a noticeable impact on the ratio of probing versus negotiated Tx link usage towards it.

### 5.3. Scenario 3: Four Nodes

**Figure 19:**
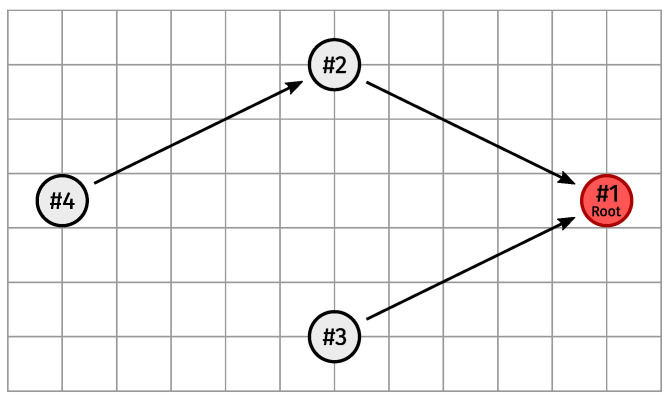
Setup of the third scenario involving four nodes, i.e., one root (#1) and three non-root nodes (#2, #3, and #4). The black arrows indicate child–parent relationships in a steady state. A single grid square measures 10 m × 10 m.

In the third scenario, we compared three configurations of the four-node setup shown in Figure 19, wherein all nodes have (1) only the sub-GHz interface-modes; (2) only interface-modes (2, 1) and (1, 1); or (3) all interface-modes listed in Table 3. We assessed the performance of these configurations (in 80 simulations each) in the face of a common adversity in industrial environments. More specifically, at a given point in time, we blocked interface-mode (2, 1) between #4 and #2 to emulate modulation-specific phenomena such as fading.

At the beginning of each simulation run, we briefly blocked all (existing) physical links from #1 to #4, between #2 and #3, and between #3 and #4 to ensure a consistent topology (i.e., following Figure 19). Additionally, we very briefly blocked interface-mode (2, 2) between #3 and #4 (should it exist) to ensure (2, 1) was #4’s frozen interface-mode towards #2. After that, the network was left to operate under normal conditions for 15 min before interface-mode (2, 1) was permanently broken between #2 and #4 at the 20 min mark (hence the reason we ensured that (2, 1) was #4’s frozen interface-mode towards #2). From there, each simulation run lasted 10 more minutes.

How a network reacts is heavily influenced by the metric normalization weights calculated using (5). As explained in [8] (Section 4.4), attaching an appropriate weight to an interface-mode becoming unavailable involves a trade-off between encouraging physical link redundancy (which discourages parent changes and promotes overall stability) and reducing the path length to the root (which reduces end-to-end latency). For the first and second configurations, we used the same parameters as in [8] (i.e., $IL_{div}=4$ and $W_{off}=0$), as each node in both configurations possessed two interface-modes. The third setup retained the parameters listed in Table 6.

For each configuration, we visualized the statistics of #4 switching to #3 as the preferred parent at any point after the 20 min mark versus #4 switching its frozen interface-mode towards #2 instead.

Before we continue, it must be noted that the rules for computing the rank to advertise (which carry over from the Minimum Rank with Hysteresis OF (MRHOF) [23] (Section 3.3)) have a considerable impact on whether and/or when #4 switches preferred parent from #2 to #3. That is, even though #2 and #3 will have approximately identical path costs towards #1, the ranks they advertise are not exactly equal. Depending on which node first receives a DIO from the other (which is completely random), #3 might end up in #2’s parent set, or vice versa. This prompts the node that has the other in its parent set to advertise a rank greater than or equal to the highest rank advertised by any member of its parent set, rounded upward to the next integral rank. In practice, this means that when #3 ends up in #2’s parent set, #3 will advertise a rank that is lower than the one #2 advertises by a non-negligible amount, making it more likely that #4 switches preferred parent from #2 to #3. In fact, in many cases where #3 is a member of #2’s parent set, the combination with the frozen interface-mode switch hysteresis threshold is enough to trigger a preferred parent switch.

**Figure 20:**
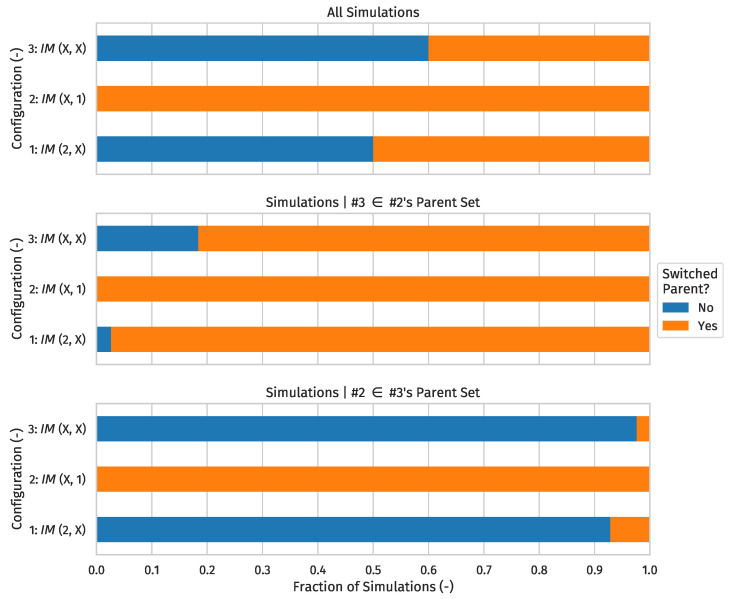
Number of times node #4 either switched preferred parent from #2 to #3 or only switched the frozen interface-mode towards #2 instead (represented as a fraction of the total number of simulations), by configuration. In the first configuration, all nodes possessed only the sub-GHz interface-modes. In the second configuration, all nodes possessed only one mode per interface, i.e., (2, 1) and (1, 1). In the third configuration, each node possessed all four interface-modes.

The data presented in Figure 20 show that the second configuration, wherein each node has only interface-modes (2, 1) and (1, 1), performed the worst. Regardless of the ranks advertised by #2 and #3, #4 always switched preferred parent from #2 to #3 eventually. That is, even if #4 first switched the frozen interface-mode towards #2, the normalized metric #4 calculated for #2 eventually became bad enough to warrant a preferred parent change, either because the inferred metric of the new frozen interface-mode (i.e., (1, 1)) increased significantly shortly after it became frozen or because of a penalty incurred due to (2, 1) becoming unavailable. This is not to say that having two interfaces with one mode each is always a bad solution. In fact, if both interface-modes had a similar range (which is, however, unlikely if both interfaces operate in different frequency ranges), it would likely perform on par with the other two configurations. Indeed, it might even outperform them in other areas, as both interface-modes are used for every simultaneous transmission (which was not the case for the first and third configurations).

At first glance, the first and third configurations performed similarly. In most cases where #2 ended up in #3’s parent set (meaning #3 advertised a higher rank than #2), and in both configurations, #4 could simply switch the frozen interface-mode towards #2 and did not need to switch preferred parent (from #2 to #3). Conversely, in many cases where #3 ended up in #2’s parent set (meaning #2 advertised a higher rank than #3), and in both configurations, #4 ultimately switched preferred parent from #2 to #3. Most often, this was due to the inferred metric of #4’s frozen interface-mode towards #2 increasing by a large enough amount such that the resulting increase in normalized metric/path cost would trigger a parent switch before #4 was allowed to switch the frozen interface-mode to #2. The slight advantage in favor of the third configuration is due to this happening somewhat less frequently with the third configuration. In any case, the first configuration would be vulnerable to wide-band interference/jamming anyway, as this would impact all communication modes operating in the same frequency range, whereas the third configuration guarantees continuity.

## 6. Conclusions

The vast communication capabilities of modern wireless devices offer several opportunities that are currently not exploited by most common implementations of IIoT architectures such as 6TiSCH. Allowing (virtual) links to be (re-)configured (at runtime) with different communication modes throughout the network would, e.g., eliminate the need for an often time-consuming/costly manual setup phase of new deployments, allow individual nodes to respond to sudden or highly localized phenomena, and potentially provide full coverage in environments where a single communication mode cannot.

Based on these opportunities and the particular implementational flexibility of the 6TiSCH link and routing layers, others have also proposed 6TiSCH-based solutions for multi-modal IIoT networks supporting dynamic link reconfiguration. However, to the best of our knowledge, these are all either based on a single fixed timeslot duration to accommodate for the slowest communication mode or (if they do support variable-length superslots) they designate individual interface-modes as RPL neighbors, as is the case with the particularly interesting work of Rady et al. [16].

To not incur the efficiency/latency penalty associated with fixed timeslot durations and to prevent the downsides of equating interface-modes to RPL neighbors (e.g., the clustering of parents belonging to a limited set of devices), we presented (in this paper) a multi-modal 6TiSCH architecture that uses variable slot duration but sees devices as RPL neighbors instead. More specifically, we proposed a combination of a newly designed decentralized TSCH SF called 3MSF and a slightly modified version of DRiPLOF [8]. As a basic proof of concept for many of the mechanisms described in this paper and to showcase the flexibility/functionality of our solution overall, we designed and evaluated several scenarios through simulations. In summary, we envision our proposed multi-modal 6TiSCH architecture as a fundamental building block for next-generation IIoT ecosystems, facilitating rapid deployment and continuous adaptation to ever-evolving industrial environments and requirements.

Lastly, in the future, we want to explore the possibility of supporting nodes with dissimilar communication capabilities, conceivably by designing mechanisms intended to manage/disseminate, e.g., the superset of available interface-modes and supported supercell lengths.

## Figures and Tables

**Table 1:** Overview of TSCH-based single-topology solutions for multi-modal Industrial Internet of Things (IIoT). Note that **✓** indicates full support, **✗** indicates no support, ± indicates partial support, and — indicates “not applicable”. Adapted from [1] with permission from the authors.

Reference	Fixed- or Variable-Duration Slots	Multi-Mode Management	Simultaneous Transmission	Tx-Only Routing Metric Support	Explicit Link Reconfiguration	6TiSCH Compliant ^a^
[13]	fixed	—	—	**✓**	—	**✓**
	variable	**✗**	**✗**	**✓**	**✗**	**✗**
[14]	fixed ^b^	**✗**	**✗**	**✓**	± ^c^	± ^d^
[16]	variable	± ^e^	**✗**	**✗**	± ^c^	**✓**
This paper	variable	**✓**	**✓**	**✓**	± ^c^	**✓**

^a^ We consider a solution compliant with 6TiSCH if it can be integrated into a 6TiSCH stack as is; ^b^ Multiple packets may be aggregated into a single timeslot; ^c^ Only (part of) unicast links are explicitly re-configurable; ^d^ 6TiSCH compliance depends on the transmission acknowledgement scheme; ^e^ Autonomous supercells (and any management traffic sent on them) are single-mode.

**Table 2:** Three-bit encoding of the TSCH mode abstraction.

*m.ii*	Value	Description
0.00	0b000 ($0_{10}$)	Legacy mode (reserved)
1.00	0b100 ($4_{10}$)	Alternating mode
0.01	0b001 ($1_{10}$)	Communication mode with index 0 of interface with index 0
1.01	0b101 ($5_{10}$)	Communication mode with index 1 of interface with index 0
0.10	0b010 ($2_{10}$)	Communication mode with index 0 of interface with index 1
1.10	0b110 ($6_{10}$)	Communication mode with index 1 of interface with index 1
0.11	0b011 ($3_{10}$)	Communication mode with index 0 of all interfaces
1.11	0b111 ($7_{10}$)	Communication mode with index 1 of all interfaces

**Table 3:** Overview of the constants used to configure the log-distance path loss model used in our simulations. Adapted from [8] with permission from the authors.

Constant	IM (1, 1)	IM (1, 2)	IM (2, 1)	IM (2, 2)
*f*	2400 MHz	2400 MHz	868 MHz	868 MHz
$P_{t}$	0 dBm	0 dBm	0 dBm	0 dBm
*S*	−100 dBm	−100 dBm	−100 dBm	−100 dBm
$RSSI_{50\%}$ ^a^	−92 dBm	−92 dBm	−92 dBm	−92 dBm
$AG_{t}$	0 dBi	0 dBi	0 dBi	0 dBi
$AG_{r}$	0 dBi	0 dBi	0 dBi	0 dBi
*c*	$3.0\times 10^{8}$ m/s	$3.0\times 10^{8}$ m/s	$3.0\times 10^{8}$ m/s	$3.0\times 10^{8}$ m/s
α	2.89	2.83	2.97	3.12
σ ^b^	3.0	3.0	5.0	5.0
$d_{ref}$	1.0 m	1.0 m	1.0 m	1.0 m
$d_{\max}$	≈118.722 m	≈131.376 m	≈207.056 m	≈160.228 m

^a^ $RSSI_{50\%}$ is only used for PDR(RSSI) calculations; ^b^ σ is only used for PL(d) calculations.

**Table 4:** Mapping of interface-modes to non-simultaneous TSCH modes, supercell lengths, and timing interval factors. Note that the timing interval factors are listed in decimal notation, but the fields of a FactorList entry follow a fixed-point binary format to save space.

Interface-mode	IM (1, 1)	IM (1, 2)	IM (2, 1)	IM (2, 2)
Data rate	250 kbps	250 kbps	100 kbps	100 kbps
**TSCH mode**	0.01	1.01	0.10	1.10
**Supercell length**	1	1	2	2
TxOffFactor	–	–	1.25	1.25
TxAckDelFactor	–	–	1.25	1.25
MaxFactor	–	–	2.75	2.75

**Table 5:** Timing interval values for a unit-duration timeslot (in microseconds). The timing intervals for supercells of length > 1 are derived from these via three factors associated with the supercell length.

Interval	Value
CcaOffset	1100 µs
Cca	128 µs
TxOffset	2120 µs
RxOffset	1020 µs
RxAckDelay	3000 µs
TxAckDelay	3400 µs
RxWait	2200 µs
AckWait	800 µs
RxTx	892 µs
MaxAck	1440 µs
MaxTx	4640 µs
Slack	400 µs
Duration	12,000 µs

**Table 6:** Overview of 6TiSCH configuration parameters common to all simulation scenarios unless noted otherwise. If a secondary value is presented in brackets, it is the 16-bit representation of that same value (as used by RPL).

Parameter	Value	Notes
MinHopRankIncrease	1 (128)	Carries over from [6]
MaxRankIncrease	7 (896)	"
MAX_PATH_COST	256 (32,768)	Carries over from [23]
MAX_LINK_METRIC	8 (1024)	"
PARENT_SWITCH_THRESHOLD	1.5 (192)	"
MaxFrameRetries	3	Carries over from [2]
MaxBe	4	"
MinBe	0	"
$ETX_{init}$	6 (768)	See Figure 12
FROZEN_SWITCH_THRESHOLD	0.5 (64)	"
*S*	8 (1024)	"
$W_{off}$	0.2	"
$IL_{max}$	3	"
$IL_{div}$	4	"
Unicast Tx buffer size	64 packets	Towards same neighbor: ≤64 normal packets, ≤4 priority packets, ≤64 packets total
MaxNumCells	64	Carries over from [7]
LimNumCellsUsedHighUp	48	Inspired by [7]
LimNumCellsUsedLowUp	16	"
LimNumCellsUsedHighDown	30	"
LimNumCellsUsedLowDown	16	"
Maximum # of concurrent 6P transactions	2	Only with different neighbors or towards the same neighbor if opposite directions and ≥1 CLEAR transaction
3MSF slotframe length	75	∀ 5 slotframes

## Data Availability

The data presented in this study are available as Supplementary Materials.

## Supplementary Materials

The full code base and curated data files are available online at https://www.mdpi.com/article/10.3390/s24082414/s1.

## Abbreviations

The following abbreviations are used in this manuscript:

3MSF	Multi-Modal Minimal Scheduling Function
6P	6TiSCH Operation Sublayer Protocol
6TiSCH	IPv6 over the TSCH mode of IEEE 802.15.4e
6top	6TiSCH Operation Sublayer
ACK	Acknowledgement
ASN	Absolute Slot Number
CBS	Current Best Supercell
CCA	Clear Channel Assessment
DIO	DODAG Information Object
DIS	DODAG Information Solicitation
DODAG	Destination-Oriented Directed Acyclic Graph
DRiPL	Dual Radio-Interface Routing Protocol for LLNs
DRiPLOF	DRiPL Objective Function
EB	Enhanced Beacon
ETX	Expected number of Transmissions
FDMA	Frequency-Division Multiple Access
FIFO	First In, First Out
GHz	Gigahertz
IE	Informational Element
IETF	Internet Engineering Task Force
IIoT	Industrial Internet of Things
IP	Internet Protocol
IPv6	IP Version 6
LLN	Low-power and Lossy Network
MAC	Medium Access Control
MHz	Megahertz
MP2P	Multi-Point to Point
MRHOF	Minimum Rank with Hysteresis Objective Function
MSB	Most Significant Bit
MSF	Minimal Scheduling Function
NUD	Neighbor Unreachability Detection
OF	Objective Function
OS	Operating System
PHY	Physical layer
PO	Parent-Oriented
RFC	Request for Comments
RPL	Routing Protocol for Low-power and Lossy Networks
Rx	Receive
SF	Scheduling Function
TDMA	Time-Division Multiple Access
TSCH	Time-Slotted Channel Hopping
Tx	Transmit

## Appendix A. Slotframe Representation

There seems to be needless confusion about TSCH slotframes, how to properly represent them, and the terminology used to describe them. However, once you realize that IEEE 802.15.4 [2] (Section 6.2.6) essentially assumes that the same node cannot simultaneously transmit and/or receive on different channels, two equally valid ways of representing a slotframe emerge. That is, it can be described in a single dimension using timeslots, with each timeslot represented by its slot offset in the slotframe only. Alternatively, it can be described in two dimensions using cells, with each cell represented by both its slot and channel offsets in the slotframe. When channel offset matters, we consider it bad practice to refer to cells as timeslots (although doing the opposite is acceptable). Moreover, there is a big difference between a two-dimensional slotframe and the result of scheduling in time-frequency space. That is, due to the channel hopping mechanism, a given channel offset translates to a different frequency channel for consecutive ASNs.

## Appendix B. Quirks of MSF

As mentioned throughout this paper, 3MSF is based on MSF [7], arguably the most popular decentralized TSCH SF. However, there are certain peculiarities to MSF. In particular, the 6P Timeout value and the windowed counter mechanism for the management of negotiated cells raise questions. That is, for a two-step transaction, according to RFC 8480 [11] (Section 3.4.4), “[t]he value of the 6P Timeout should be greater than the longest possible time it takes to receive the 6P Response”. MSF then tries to fulfill this requirement by defining the 6P Timeout according to (A1).

(A1) $$\left(2^{MaxBe}-1\right)\times MaxFrameRetries\times \left|SO\right|$$

where:

MaxBe	the largest backoff exponent used by the TSCH backoff algorithm for shared cells [2] (Section 6.2.5.3);
MaxFrameRetries	the maximum number of retransmissions of a single packet, which equates to the maximum number of Tx attempts − 1;
|SO|	the number of slot offsets in a slotframe (starting from 0), i.e., the “slotframe length”.

At first glance, this accounts for the worst-case scenario, wherein the transmission of a 6P response is only successful at the last possible Tx attempt, all those attempts occurred on shared cells, and we had to back off for the largest possible number of shared cells in between them. However, this fails to account for any sort of buffering of outgoing packets, which is part of most (if not all) TSCH implementations since opportunities to transmit towards a given neighbor can be scarce.

This ties in with MSF’s second quirk, i.e., the windowed counter mechanism for negotiated cell management. More specifically, the mechanism for adding/deleting negotiated Rx cells towards the preferred parent is slightly problematic. That is, unlike a negotiated Tx cell, immediately adding at least one negotiated Rx cell towards a parent when it becomes preferred is not mandatory. Instead, MSF [7] says to count incoming packets (from the preferred parent) on the autonomous Rx cell when a node has no negotiated Rx cell towards its preferred parent yet and to add one such cell when the count (for those incoming packets) exceeds a certain threshold in a given counter window. However, autonomous cells are shared, and the concept of backing off only applies to Tx cells, meaning the backoff mechanism is transparent to the node at the Rx side of a shared cell! As such, if there is a lot of contention on a given autonomous cell, a parent might back off an excessive number of times towards a given child. Upon this occurrence, the child might erroneously understand there to be no need for a dedicated negotiated Rx cell towards its preferred parent while the parent’s outgoing buffer keeps filling up with packets, eventually causing it to overflow. To address this issue, we propose using a lower value for LIM_NUMCELLSUSED_HIGH before adding the first negotiated Rx cell towards the preferred parent and using the normal limit value thereafter, even if the first negotiated Rx cell is subsequently removed. It should be noted that 3MSF does not use this bootstrap threshold because it does not rely on shared cells in any way.

Addressing the issue with the 6P Timeout value is less straightforward. Assuming transmit buffers have a simple First In, First Out (FIFO) structure, if we tried to increase the value to accommodate any meaningful buffer size (i.e., by multiplying (A1) by the buffer size, assuming the 6P response is always at the back of the buffer), the timeout would (very) quickly become so large that 6top would become virtually unusable (since there is a typically rather low limit to the number of simultaneously ongoing 6P transactions any single node can support) and the network would grind to a halt, which is not so far-fetched if you consider that most 6P responses are sent on shared (!) autonomous cells. Short of drastic measures like, e.g., not allowing any retransmissions (which is entirely unreasonable), we can only think of an (imperfect) solution whereby the transmit buffers (towards unicast neighbors) are logically divided into a “priority” buffer (for 6P and other important traffic) and a normal buffer (for all other traffic), allowing important traffic to skip the queue, so to speak. This, in turn, would require MAC sequence numbers to be assigned only when transmission of a packet is first attempted, and so on.

## Appendix C. RPL Management Traffic Rules

Since supercells and not packets are now tagged (through a TSCH mode) with the interface-mode(s) to use for their transmission, all the rules of DRiPLOF concerning RPL management traffic [8] (Section 4.1) are forfeited. That said, broadcast RPL messages must still be sent on all interface-modes. More specifically, an identical copy of the same broadcast RPL message must be sent on as many consecutive minimal supercells as there are minimal supercells. However, this does not necessarily have to happen in the same slotframe. This could be achieved by, e.g., adding as many copies of a given message to the broadcast buffer as there are minimal supercells, assuming the broadcast buffer is a FIFO buffer.
