# Supplementary material for: 3MSF: A Multi-Modal Adaptation of the 6TiSCH Minimal Scheduling Function for the Industrial IoT
Source: Sensors (Basel). 2024 Apr 10;24(8):2414. doi: 10.3390/s24082414 (PMC11054304; doi:10.3390/s24082414)
Supplement: Supplementary file 1 [file sensors-24-02414-s001.zip › code/instructions.pdf]

Multi-Modal Minimal Scheduling Function (3MSF)

This document describes the usage of the twofaced example for Contiki-NG. It is designed to work with the Cooja simulator. The purpose of the twofaced application is to evaluate our implementation of a multi-modal 6TiSCH architecture based on a newly designed TSCH SF named 3MSF and a slightly modified version of DRiPLOF, a RPL OF we designed previously. If you make use of this example or any of its parts in your own research, we ask you to include (in your own research output) a reference to the paper accompanying this example:

"3MSF: A Multi-Modal Adaptation of the 6TiSCH Minimal Scheduling Function for Industrial IoT" by R. Elsas, D. Van Leemput, E. De Poorter, and J. Hoebeke (in submission)

More information on DRiPLOF can be found in:

"DRiPLOF: An RPL Extension for Multi-Interface Wireless Sensor Networks in Interference-Prone Environments" by R. Elsas, E. De Poorter, and J. Hoebeke

Table of Contents

- Multi-Modal Minimal Scheduling Function (3MSF)
  - Table of Contents
  - Getting Started
  - Usage
    - Project Structure & Configuration
      - Physical Layer
      - Link Layer
      - Network Layer
    - Simulation
      - Cooja
      - Experiments
      - Log Parsing & Visualization
  - Recommended Resources
  - Contact and Affiliation
  - Licensing

Getting Started

The first step is to clone our remote repository to your local machine, updating all git submodules and checking out the `tdripl` branch as follows:

```
$ cd ~
$ git clone https://github.com/tinstructor/contiki-ng.git
$ cd ~/contiki-ng/
$ git submodule update --init --recursive
$ git checkout tdripl
```

If you'd like to adopt the proper git workflow (i.e., the forking workflow) for this project, according to the Atlassian git workflow tutorial, you'd first need to create

your own remote repository (often named `upstream`) and local branch before pushing that branch to your remote and setting the newly created local branch to track the corresponding branch of the remote. The following code snippet shows how this is done.

> ***Note:*** *there are shorter ways to achieve this, but the following snippet breaks it down into easy steps, sort off.*

```
$ cd ~/contiki-ng/
$ git remote add upstream <link to your remote>
$ git checkout -b <new branch>
$ git push upstream <new branch>
$ git branch -u upstream/<new branch>
```

The next step is to install Docker. The installation process (for Ubuntu) starts by making sure there are no older versions of Docker installed. Next, it is recommended to install Docker through its repository as explained here. Following the installation of Docker, you must first make sure your user is added to the `docker` group and rebooting as follows:

```
$ sudo usermod -a -G docker <user>
$ sudo reboot
```

Then, you can pull the following Docker Contiki-NG image:

```
$ docker pull contiker/contiki-
ng:cce382ff29d4569f7b9b2e7863ec1e8e49221e92dced263b001ba2ba26408170
```

Now open up `.bashrc`:

```
$ cd ~
$ sudo nano .bashrc
```

Append the following lines to the end of the file:

```
export CNG_PATH="$PATH:$HOME/contiki-ng"
alias contiker="docker run --privileged --sysctl net.ipv6.conf.all.disable_ipv6=0 --
mount type=bind,source=$CNG_PATH,destination=$PATH:$HOME/contiki-ng -e
DISPLAY=$DISPLAY -v /tmp/.X11-unix:/tmp/.X11-unix -v /dev/bus/usb:/dev/bus/usb -ti
contiker/contiki-ng"
```

and run the following command:

```
$ exec bash
```

FINALLY you can start making changes to the code base. The twofaced application is located in `contiki-ng > examples > twofaced`. You might want to download VSCode as a convenient IDE for browsing through the code base. It comes with features that are very usefull when coding, especially in terms of code completion.

> ***Note:*** *for more information on the structure of the code base and how to modify it, have a look at the Contiki-NG Docs.*

Usage

This example may be used to achieve several goals, each of which implies a slightly different way in which to use it. Nonetheless, in any case, compiling this example for a certain (supported) platform results in the creation of binaries for two different kinds of RPL nodes: non-root nodes (as implemented in `twofaced-node.c`) and root nodes (as implemented in `twofaced-root.c`).

> ***Note:*** *manually compiling is of little importance to you if you only wish to run a Cooja simulation. Nonetheless, it is probably a good idea to have a basic understanding of how this works.*

Project Structure & Configuration

This project was written to enable trully multi-modal 6TiSCH operation. In order for that to work, several additions / changes needed to be made to the Contiki-NG code base. Many of these changes closely align with the 3 bottom layers of the TCP / IP stack.

Physical Layer

We modified `arch/platform/cooja/dev/cooja-radio.c` and `arch/platform/cooja/dev/cooja-radio.h` (i.e., the Cooja radio driver). This driver manipulates / reads certain variables which are then accessed by the Cooja simulator through JNI.

> *More information available upon request.*

Link Layer

Contrary to the generally accepted process for defining a new MAC layer in Contiki-NG, we do not set the `MAKE_MAC` make variable to `MAKE_MAC_OTHER` in `examples/twofaced/Makefile`. Instead, we do the following:

```
MAKE_MAC = MAKE_MAC_TSCHFACED

ifeq ($(MAKE_MAC),MAKE_MAC_TSCHFACED)
  MODULES_REL += net/mac/twofaced-tsch
  CFLAGS += -DMAC_CONF_WITH_TSCHFACED=1
  MODULES += os/net/mac/tsch/sixtop
  MODULES += $(CONTIKI_NG_SERVICES_DIR)/msf
endif
```

This allows us to perform preprocessor checks on `MAC_CONF_WITH_TSCHFACED` rather than `MAC_CONF_WITH_OTHER` and includes the module required to run the twofaced TSCH MAC protocol only when necessary. This MAC protocol is an adaptation of the TSCH MAC protocol with some multi-modal specific changes / additions. Note that when `MAKE_MAC` is set to `MAKE_MAC_TSCHFACED` (in `examples/twofaced/Makefile`), the contents of the `examples/twofaced/net/mac/twofaced-tsch` directory are included in the build path. The files in this directory make up the twofaced TSCH MAC protocol (the driver is in there somewhere). In addition, a `CFLAG` is added. This ensures that the macro `MAC_CONF_WITH_TSCHFACED` is defined and set to `1` accross the whole of Contiki-NG (which is pretty usefull for performing some checks) prior to any other macros being defined (at least those that aren't passed as `CFLAG`). As such, `examples/twofaced/project-conf.h` allows us to automatically set the MAC driver to `twofaced_tsch_driver` when setting `MAKE_MAC` to `MAKE_MAC_TSCHFACED`, because doing so includes the correct driver files and defines `MAC_CONF_WITH_TSCHFACED` (this is pretty nifty stuff innit):

```
#if MAC_CONF_WITH_TSCHFACED
...
#define NETSTACK_CONF_MAC twofaced_tsch_driver
...
#endif /* MAC_CONF_WITH_TSCHFACED */
```

The MAC driver interface for the twofaced TSCH MAC (i.e., `twofaced_tsch_driver`) is defined in `examples/twofaced/net/mac/twofaced-mac/twofaced-tsch.c`.

> ***Note:*** *the functions pointed to by the MAC driver can be accessed through the `NETSTACK_MAC` macro. For example, the selected MAC protocol is initialized in `os/net/netstack.c` by calling `NETSTACK_MAC.init()`.*

```
const struct mac_driver twofaced_tsch_driver = {
  "twofaced_mac",
  init,
  send,
  input,
  on,
  off,
  max_payload,
  lock_input,
  unlock_input,
};
```

> *More information available upon request.*

This brings us to the way in which Contiki-NG keeps statistics about links to neighbors. More specifically, it keeps track of per-neighbor link-statistics by storing value entries of type `struct link_stats` in a Contiki-NG `NBR_TABLE` called `link_stats` (see `os/net/link-stats.c`). Upon every valid Tx attempt of a (unicast) packet, the function `link_stats_packet_sent()` is called (defined in `os/net/link-stats.c` and declared in `os/net/link-stats.h`). Here, the link_stats table is checked for an existing entry keyed on the link-layer address of the unicast neighbor to which Tx was attempted. If no existing entry is found, a new entry in the link_stats neighbor table is added. If adding the new entry was succesful, a linked-list (consisting of structs of a certain format, see `os/lib/list.h`) named `interface_list`, which is a member of a `link_stats` struct object (and thus of each link-stats neighbor table entry), can be initialized.

Next, whether or not the entry was newly added doesn't really matter, the `interface_list` of the given link-stats table entry (i.e., the one we found or otherwise newly added just now) is checked for an entry (of the type `struct interface_list_entry`) containing the identifier (this is NOT a MAC address) of the interface that was put in the packetbuffer attribute `PACKETBUF_ATTR_INTERFACE_ID` by the twofaced TSCH MAC. If such entry was found (i.e., `interface_list_entry_from_id()` didn't return `NULL`), the existing interface list entry is updated. Otherwise, when no interface list entry is found for the given interface identifier, a new entry is added if appropriate. When a new interface list entry is created, a linked-list (consisting of structs of a certain format, see `os/lib/list.h`) named `mode_list`, which is a member of an `interface_list_entry` struct object (and thus of each interface list entry, which itself is in turn part of a link-stats neighbor table entry), can be initialized.

Mode list entries work in similar fashion to the interface-list entries they are part of. That is, when there is no entry for a mode with a given id in a mode_list of a particular interface list entry, a new mode list entry is added if appropriate. In summary, an entry in the link stats table corresponds to a neighbor, each link stats table entry contains an interface list of which each entry corresponds to a given interface of a given neighbor, and each interface list entry contains a mode list of which each entry hence corresponds to a given mode on a given interface of a given neighbor. Mode list entries store all the important information (i.e., for an interface-mode of a given neighbor) such as the stored inferred metric, tx and ack counters, the noack counter, and so on. The normalized metric corresponding to a given neighbor, as well as a reference to the preferred and (potentially) frozen (i.e., if the neighbor is the preferred parent) interface-mode towards said neighbor, is kept in the link stats table entry (i.e., on the same level as the interface list).

> ***Note:*** *a Contiki-NG `NBR_TABLE` is pretty straightforward once you get the hang of it. Have a look at `os/net/nbr-table.h`. If you don't speak fluent preprocessor hocus-pocus, don't worry. What it boils down to is that each entry in a neighbor table is keyed on the link-layer address of a neighbor and stores entries of a given type.*

> ***Note:*** *a similar function named `link_stats_input_callback()` is called when a packet is received from a neighbor. Since the functionality provided by this function with regard to multi-modal operation is almost (not completely) identical to `link_stats_packet_sent()` you should be able to understand how it works by just looking at `os/net/link-stats.c`. Hence, we shall not discuss it any further.*

The inferred metric stored in mode list entries is used for, amongst other things, selecting the preferred interface-mode towards a given neighbor (i.e., the neighbor represented by a link-stats table entry), calculating the normalized metric towards that same neighbor, and so on. Preferred interface-mode selection is handled by the function `link_stats_select_pref_interface_and_mode()` in `os/net/link-stats.c`. The normalized metric of a neighbor is updated by calling `link_stats_update_norm_metric()` in `os/net/link-stats.c` (an action that is exclusively coordinated by the routing layer).

By default `get_valid_link_norm()` is used for calculating the normalized metric of a neighbor. It accepts, as a function parameter, the inferred metric of the preferred interface-mode towards a given neighbor (or of the frozen interface-mode towards the preferred parent), together with the number of available interface-modes towards that same neighbor:

```
uint16_t
get_valid_link_norm(uint16_t preferred_metric, uint8_t valid_links)
{
  if(LINK_STATS_WORSE_THAN_THRESH(preferred_metric) ||
     valid_links > num_req_modes_total ||
     valid_links == 0) {
    return 0xffff;
  }
  ...
  uint8_t invalid_links = num_req_modes_total - valid_links;
  if(invalid_links > LINK_STATS_MAX_INVALID_LINKS) {
    return 0xffff;
  }
```

```
  ...
  double weight = (MIN(invalid_links, LINK_STATS_MAX_INVALID_LINKS) *
LINK_STATS_INVALID_LINKS_DIV_BASE) / (double)LINK_STATS_INVALID_LINKS_DIV;
  ...
  if(weight > 0) {
    weight -= LINK_STATS_WEIGHT_OFFSET;
  }
  return weight * LINK_STATS_SCALE_MULTIPLIER + preferred_metric * (1.0 - weight) +
0.5;
}
```

> *More information available upon request.*

Network Layer

This layer is very similar to the routing layer we implemented for our prior work on:

"DRiPLOF: An RPL Extension for Multi-Interface Wireless Sensor Networks in Interference-Prone Environments" by R. Elsas, E. De Poorter, and J. Hoebeke

> *More information available upon request.*

Simulation

Cooja

Since we've made some modifications to Cooja for dual-interface support, this is where you change out the default Cooja implementation included with Contiki-NG for our implementation. You can do this by means of some Git submodule hocus pocus:

```
$ cd ~/contiki-ng
$ git config --file=.gitmodules submodule.tools/cooja.url
https://github.com/tinstructor/cooja.git
$ git config --file=.gitmodules submodule.tools/cooja.branch dripl
$ git submodule sync
$ git submodule update --init --recursive
$ cd tools/cooja/
$ git fetch
$ git checkout dripl
$ cd ../..
```

> ***Note:*** *It's probably a good idea to make a custom command to perform this task repeatedly.*

If you want to change back to the default Cooja implementation, you can run:

```
$ cd ~/contiki-ng
$ rm -rf tools/cooja
$ git checkout HEAD -- .gitmodules
$ git submodule update --init --recursive
$ git submodule sync
$ git submodule update --init --recursive
$ cd tools/cooja/
$ git remote prune origin
```

```
$ git branch -D dripl
$ cd ../..
```

> ***Note:*** *It's probably a good idea to make a custom command to perform this task repeatedly.*

You are now ready to start simulating with Cooja!

Experiments

To run a string of simulations (of our 6TiSCH architecture) you do not need to fiddle with Cooja yourself whatsoever, nor do you have to touch any configuration files. Instead, we have provided a python script that, according to the information you give it / configure it with, automatically modifies all necessary files (i.e., source files, makefiles, simulation files and scripts, and so on) and performs a given number of simulation runs (each with a unique random seed) of a specified duration by repeatedly running Cooja in headless mode. That is, the `tschfaced_simrunner.py` script in `examples/twofaced/python/` takes, as an (optional) argument, an index into a list of which each entry is itself a list of configuration parameters. More specifically, the `scenario_lists` in `tschfaced_simrunner.py` is structured as a list of lists. Each inner list represents a string of simulations, and each element within the inner list corresponds to a specific parameter for said string of simulations:

```
parser = argparse.ArgumentParser()
parser.add_argument("--scenario", type=int, help="Simulation scenario.")
args = parser.parse_args()

# other stuff

# Interface-mode flags:
#     - 0b0001 = primary interface and primary mode
#     - 0b0010 = primary interface and secondary mode
#     - 0b0100 = secondary interface and primary mode
#     - 0b1000 = secondary interface and secondary mode

# the following is just an example, the actual list is different
scenario_lists = [
    [   # Scenario 0
        [],                         # List of mote IDs to comment out
        1,                          # Number of simulations
        602,                        # Simulation duration (in seconds)
        300,                        # Simulation hangtime (in seconds)
        False,                      # Interfere same mode only flag
        True,                       # TSCH CCA off flag
        [],                         # Move parameters
        [],                         # Jam parameters
        [],                         # Break parameters
        0b1111                      # Interface mode configuration
    ],
    [   # Scenario 1
        list(range(1, 18)),          # List of mote IDs to comment out
        40,                          # Number of simulations
        1202,                        # Simulation duration (in seconds)
        60,                          # Simulation hangtime (in seconds)
```

```
        False,                          # Interfere same mode only flag
        True,                           # TSCH CCA off flag
        [],                             # Move parameters
        [],                             # Jam parameters
        [                               # Break parameters
            (18, 600, 603, 19, 0b1100),
            (19, 600, 603, 18, 0b1100)
        ],
        0b1111                          # Interface mode configuration
    ],
    # Other scenarios follow...
]

scenario = 0
if args.scenario is not None and args.scenario in range(0, len(scenario_lists)):
    scenario = args.scenario

# rest of code
```

Note that all simulations that can be run use some variation of the simulation setup specified by `examples/twofaced/tschfaced.csc`. Each string of simulations is represented by a list with the following elements:

1. **List of mote IDs to comment out**: This is a list of integer IDs corresponding to motes that should be commented out in the simulation configuration file (i.e., `examples/twofaced/tschfaced.csc`).
2. **Number of simulations**: Indicates how many simulations to run in this string of simulations (and with the specified configuration).
3. **Simulation duration**: Specifies the duration of each simulation in seconds.
4. **Simulation hangtime**: Represents the max hangtime of each simulation in seconds (each simulation spawns a new subprocess and this hangtime specifies the maximum amount of seconds a subprocess can not create any output before it is killed and respawned).
5. **Interfere same mode only flag**: not relevant for our paper because we don't use an interfering node but break links instead
6. **TSCH CCA off flag**: Specifies whether TSCH CCA (Clear Channel Assessment) is turned off (True or False), was always false for the simulations we ran.
7. **Move parameters**: This is a list of tuples representing parameters for moving motes specified in the simulation configuration file (i.e., `examples/twofaced/tschfaced.csc`), provided they have not already been commented out. Each tuple contains information about the mote ID, time, and coordinates for moving the mote.
8. **Jam parameters**: not relevant for our paper because we don't use an interfering node but break links instead
9. **Break parameters**: Contains parameters for breaking links in the simulation. Each tuple consists of the node ID, break start time, break duration, peer ID, and interface-mode flags. The interface-mode flags point to the interface-modes accross which no packets that are sent by the node with node ID shall arrive at the node with peer ID for the break duration and starting at the specified time.
10. **Interface mode configuration**: Represents the configuration of interface-modes (for all nodes of a simulation) using a binary flag. That is, each node shall be configured to have only the interface-modes for which the flag is set.

To perform an actual string of simulations you have to first swap out the Cooja implementation for our implementation as follows:

```
$ cd ~/contiki-ng
$ git config --file=.gitmodules submodule.tools/cooja.url
https://github.com/tinstructor/cooja.git
$ git config --file=.gitmodules submodule.tools/cooja.branch dripl
$ git submodule sync
$ git submodule update --init --recursive
$ cd tools/cooja/
$ git fetch
$ git checkout dripl
$ cd ../..
```

Then, remove any old docker containers:

```
$ docker container prune
```

Next, open a new docker container and within it, execute the python script as follows:

```
$ contiker
To run a command as administrator (user "root"), use "sudo <command>".
See "man sudo_root" for details.

user@c6608370d0d4:~/contiki-ng$ cd examples/twofaced/python/
user@c6608370d0d4:~/contiki-ng/examples/twofaced/python$ python3
tschfaced_simrunner.py --scenario 0
```

When a string of simulations concludes, it is best to exit the docker container, prune it, and then start a new one should you wish to perform another string of simulations:

```
user@c6608370d0d4:~/contiki-ng/examples/twofaced/python$ exit
logout
$ docker container prune -y
$ contiker
To run a command as administrator (user "root"), use "sudo <command>".
See "man sudo_root" for details.

user@c6608370d0d4:~/contiki-ng$ cd examples/twofaced/python/
...
```

Running `python3 tschfaced_simrunner.py --scenario 1` in the docker container will cause the following parameter list to be used:

```
scenario_lists = [
                  ...
                  [list(range(1,18)), 40, 1202, 60, False, True, [], [], [(18, 600,
603, 19, 0b1100), (19, 600, 603, 18, 0b1100)], 0b1111],
                  ...
                 ]
```

which corresponds to scenario 1 of our paper. That is, we use only 2 nodes (node with id 18, which is a non-root node that maps to node #2 in scenario 1 of our paper, and

node with id 19, which is the root node corresponding to node #1 in scenario 1 of our paper). We run 40 simulations with this exact configuration (each with a different random seed though), each simulation lasts 1202 seconds, all nodes have all interface-modes, and at the 10-minute mark all communication modes of the secondary interface (which is the sub-GHz interface) are broken in both directions for the remainder of the simulation run.

Similarly running `python3 tschfaced_simrunner.py --scenario 2` in the docker container will cause the following parameter list to be used:

```
scenario_lists = [
                  ...
                  [list(range(1,16)) + [17], 40, 1202, 80, False, True, [], [], [],
0b1111],
                  ...
                 ]
```

which corresponds to scenario 2 of our paper. That is, we use 3 nodes (node with id 16, which is a non-root node that maps to node #3 in scenario 2 of our paper, node with id 18, which is a non-root node that maps to node #2 in scenario 2 of our paper, and node with id 19, which is the root node corresponding to node #1 in scenario 2 of our paper). We run 40 simulations with this exact configuration (each with a different random seed though), each simulation lasts 1202 seconds, and all nodes have all interface-modes.

Scenario 3 of our paper is split into 3 different configurations. Running `python3 tschfaced_simrunner.py --scenario 3` in the docker container corresponds to the string of simulations required for configuration 1 of scenario 3, i.e., wherein all nodes are configured to have only the sub-GHz interface-modes. Running `python3 tschfaced_simrunner.py --scenario 4` in the docker container corresponds to the string of simulations required for configuration 2 of scenario 3, i.e., wherein all nodes are configured to have only interface-mode (2,1) and (1,1). Running `python3 tschfaced_simrunner.py --scenario 5` in the docker container corresponds to the string of simulations required for configuration 3 of scenario 3, i.e., wherein all nodes are configured to have all 4 interface-modes.

Performing a string of simulations causes an appropriately named logfile for every simulation of a given simulation string to be written to `examples/twofaced/logs/`. If you rerun a string of simulations with the same index into the `scenario_lists`, all existing logfiles for said string of simulations are first removed from this directory (meaning no manual cleanup is required). For example, if you run the string of simulations with index one, you should see something like the following accumulation of logfiles in the `examples/twofaced/logs/` folder:

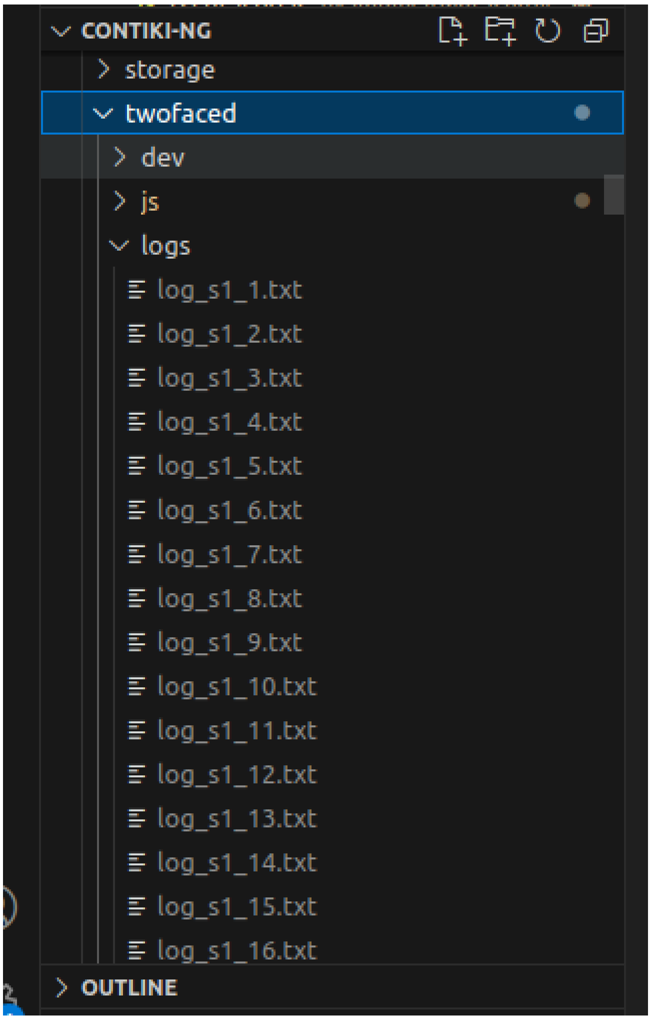

> *More information available upon request.*

Log Parsing & Visualization

Once you have ran a string of simulations to completion and all the corresponding logfiles have been generated, you can now parse all logfiles belonging to a given string of simulations and store all relevant information in an intermediate non-human-readable format by doing something like the following (does not have to be in the docker container anymore):

```
$ cd ~/contiki-ng/examples/twofaced/python/
$ python3 tschfaced_logparser.py 1 ../logs/ ../pickles/
```

which in this case will parse all logs of the string of simulations with index one (contained in `examples/twofaced/logs/`, this corresponds to scenario 1 of our paper) and will store all relevant information pertaining to this string of simulations in a serialized data structure to be stored in `examples/twofaced/pickles/`. This command works in identical fashion for parsing all logfiles pertaining to the string of simulations with index 2 (which corresponds to scenario 2 of our paper), the string of simulations with index 3 (which corresponds to configuration 1 of scenario 3 of our paper), the string of simulations with index 4 (which corresponds to configuration 2

of scenario 3 of our paper), and the string of simulations with index 5 (which corresponds to configuration 3 of scenario 3 of our paper).

Once you have generated the intermediate data structure for all strings of simulations you should get something like the following:

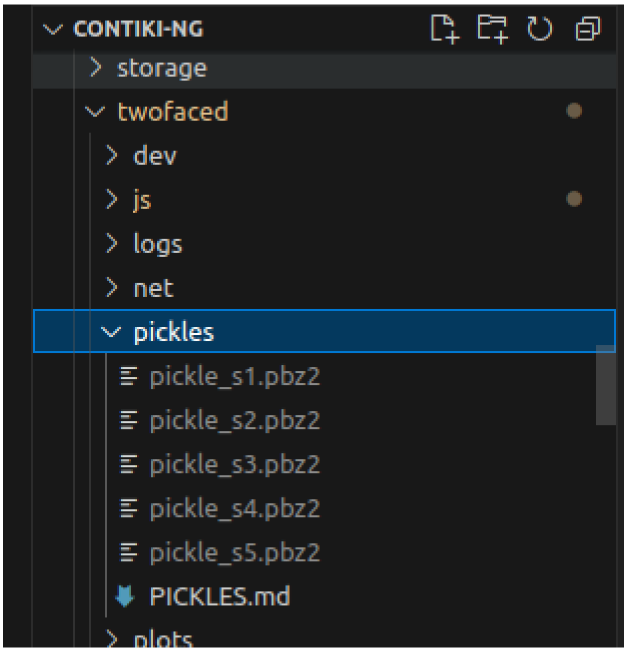

Finally, you can generate all the plots corresponding to the 3 scenarios of our paper using the `examples/twofaced/python/tschfaced_pickleparser.py` script. More specifically, if you wish to generate all the plots corresponding to scenario 1 of our paper and store them as pdf files, you can run the following commands (does not need to be in the docker container anymore):

```
$ cd ~/contiki-ng/examples/twofaced/python/
$ python3 tschfaced_pickleparser.py 1 ../pickles/ --plot_dir ../plots/
```

If you wish to do the same for scenario 2 of our paper you can run:

```
$ cd ~/contiki-ng/examples/twofaced/python/
$ python3 tschfaced_pickleparser.py 2 ../pickles/ --plot_dir ../plots/
```

Finally, generating the plots for scenario 3 of our paper is as easy as running:

```
$ cd ~/contiki-ng/examples/twofaced/python/
$ python3 tschfaced_pickleparser.py 3 ../pickles/ --plot_dir ../plots/
```

The latter would have also worked if you had specified index 4 or 5, which all result in the same output (as any of these 3 commands will look at all 3 intermediate data structures corresponding to the configurations of scenario 3 of our paper).

In the end, you should be left with the following:

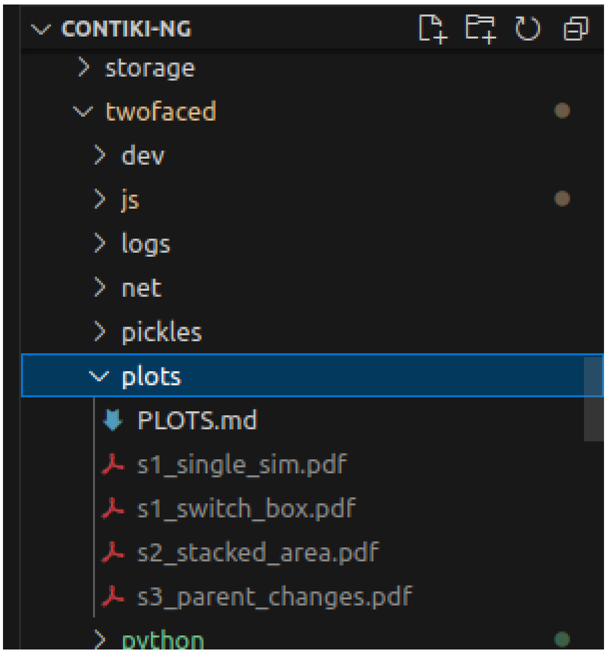

> ***Note:*** *that parsing all the logfiles of a given string of simulations takes a considerable amount of time. As such, parsing all the logfiles of a given string of simulations only once and storing all relevant information in an intermediate format which is much faster to operate on allowed us to design the visualizations without having to wait an enternity every time we made a small adjustment.*

> *More information available upon request.*

Recommended Resources

- **The Contiki-NG Docs**: It is generally advisable to start out here. However, the documentation is really basic and leaves much to be desired, especially if you want to do more than just write applications on top of existing functionality.
- **Practical Contiki-NG** *by Agus Kurniawan*: After you've gone through the Wiki you'll have a lot of unanswered questions about how stuff actually works. This book can help you with a small part of those questions, but don't expect an in-depth explanation of anything PHY related. If you have a Shibboleth or Athens account via your research institution, you can even download a digital copy for free, legally!
- **IEEE 802.15.4-2020**: The currently active PHY + MAC layer standard for 802.15.4 networks. Although this is the official standard, many developers seem to have a total disregard for certain aspects of it. Especially on the Sub-GHz PHY layers, there seems to be a lot of confusion as to what is actually standardised and what is not. The fact that IEEE standards are not publicly available and otherwise very expensive to obtain doesn't help this confusion either.
- **Jump Start Git** *by Shaumik Daityari*: As mentioned before, if you don't speak Git, this book is where you might want to start your journey.
- **C in a Nutshell** *by Peter Prinz & Tony Crawford*: Although C is an incredibly forgiving language when it comes to getting what you want out of it (hence the abundance of terribly written but otherwise "functional" code), some parts of

our source code contain more advanced features and contstructs from the C99 and later C11 specification. We make no mistake about it, seasoned embedded developers would probably have a heart-attack looking at parts of our code, but the important thing to remember is that we're well on our way to write better source code and this book is what's getting us there.
- **The Java Programming Masterclass** *by Tim Buchalka*: want to learn modern Java programming with minimal to no experience in Object-Oriented Programming (OOP)? Just looking for a refresher on OOP best-practices and / or the Java programming language because it has been a while? Whatever your skill- and / or comfort level, this beautifully structured online course will get you there in no-time (provided you are willing to put in the work). Happy hunting! (This course is especially useful if you want to understand / modify the Cooja project)

Contact and Affiliation

Robbe Elsas

email: robbe.elsas@ugent.be

affiliation: IDLab Ghent University - imec

Licensing

Unless explicitly noted otherwise, all files belonging to this example are licensed under a BSD 3-Clause license:

> *Copyright (c) 2024, Ghent University. All rights reserved.*
>
> *Redistribution and use in source and binary forms, with or without modification, are permitted provided that the following conditions are met:*
>
> 1. *Redistributions of source code must retain the above copyright notice, this list of conditions and the following disclaimer.*
> 2. *Redistributions in binary form must reproduce the above copyright notice, this list of conditions and the following disclaimer in the documentation and/or other materials provided with the distribution.*
> 3. *Neither the name of the Institute nor the names of its contributors may be used to endorse or promote products derived from this software without specific prior written permission.*
>
> *THIS SOFTWARE IS PROVIDED BY THE INSTITUTE AND CONTRIBUTORS "AS IS" AND ANY EXPRESS OR IMPLIED WARRANTIES, INCLUDING, BUT NOT LIMITED TO, THE IMPLIED WARRANTIES OF MERCHANTABILITY AND FITNESS FOR A PARTICULAR PURPOSE ARE DISCLAIMED. IN NO EVENT SHALL THE INSTITUTE OR CONTRIBUTORS BE LIABLE FOR ANY DIRECT, INDIRECT, INCIDENTAL, SPECIAL, EXEMPLARY, OR CONSEQUENTIAL DAMAGES (INCLUDING, BUT NOT LIMITED TO, PROCUREMENT OF SUBSTITUTE GOODS OR SERVICES; LOSS OF USE, DATA, OR PROFITS; OR BUSINESS INTERRUPTION) HOWEVER CAUSED AND ON ANY THEORY OF LIABILITY, WHETHER IN CONTRACT, STRICT LIABILITY, OR TORT (INCLUDING NEGLIGENCE OR OTHERWISE) ARISING IN ANY WAY OUT OF THE USE OF THIS SOFTWARE, EVEN IF ADVISED OF THE POSSIBILITY OF SUCH DAMAGE.*
