# Supplementary material for: 3MSF: A Multi-Modal Adaptation of the 6TiSCH Minimal Scheduling Function for the Industrial IoT
Source: Sensors (Basel). 2024 Apr 10;24(8):2414. doi: 10.3390/s24082414 (PMC11054304; doi:10.3390/s24082414)
Supplement: Supplementary file 1 [file sensors-24-02414-s001.zip › data/plots/s1_single_sim.pdf]

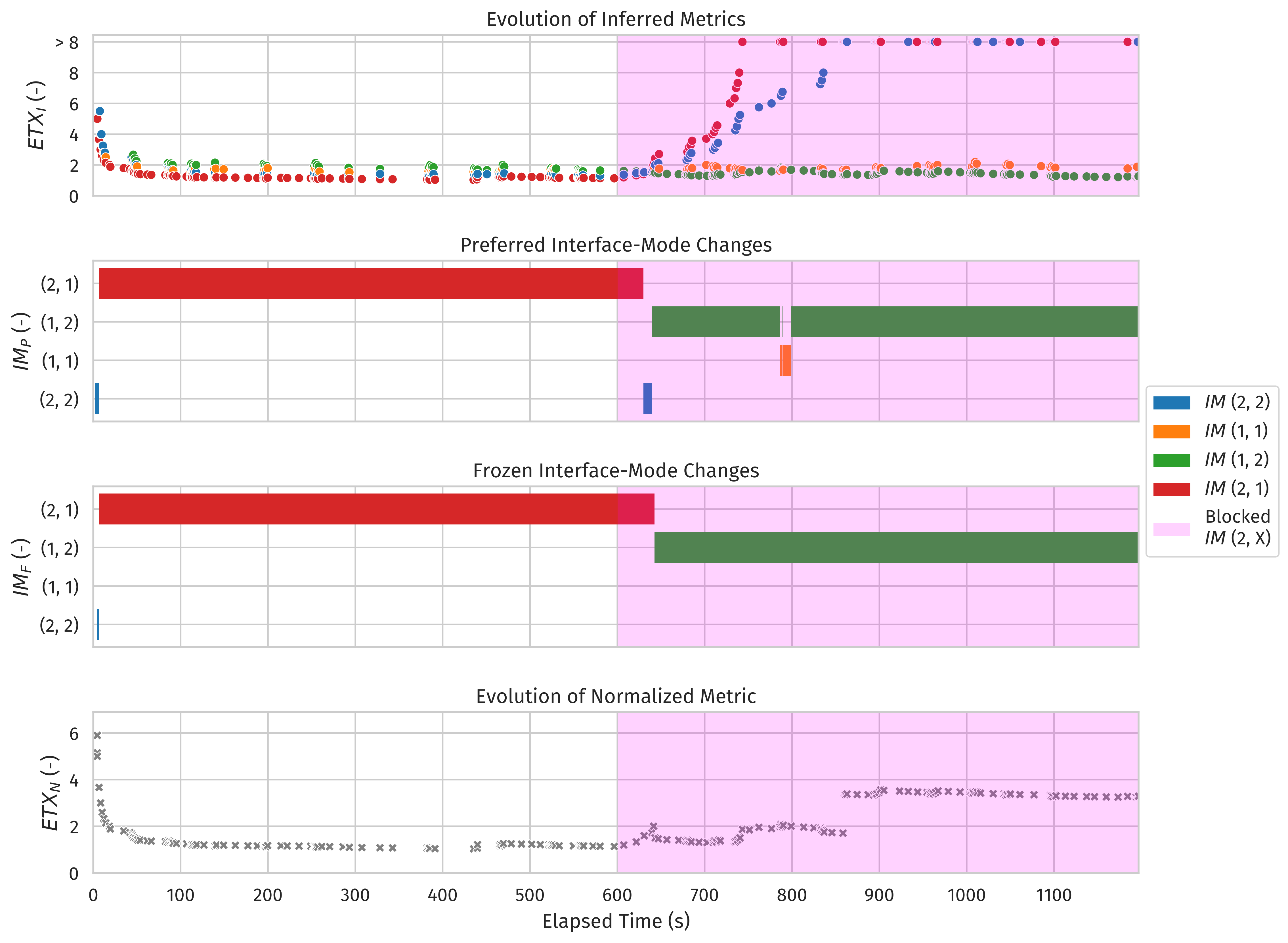

Evolution of Inferred Metrics
> 8
8
6
4
2
0
ETX_I (-)
Preferred Interface-Mode Changes
(2, 1)
(1, 2)
(1, 1)
(2, 2)
IM_P (-)
Frozen Interface-Mode Changes
(2, 1)
(1, 2)
(1, 1)
(2, 2)
IM_F (-)
Evolution of Normalized Metric
6
4
2
0
ETX_N (-)
0
100
200
300
400
500
600
700
800
900
1000
1100
Elapsed Time (s)
IM (2, 2)
IM (1, 1)
IM (1, 2)
IM (2, 1)
Blocked
IM (2, X)
