# Supplementary material for: 3MSF: A Multi-Modal Adaptation of the 6TiSCH Minimal Scheduling Function for the Industrial IoT
Source: Sensors (Basel). 2024 Apr 10;24(8):2414. doi: 10.3390/s24082414 (PMC11054304; doi:10.3390/s24082414)
Supplement: Supplementary file 1 [file sensors-24-02414-s001.zip › data/plots/s1_switch_box.pdf]

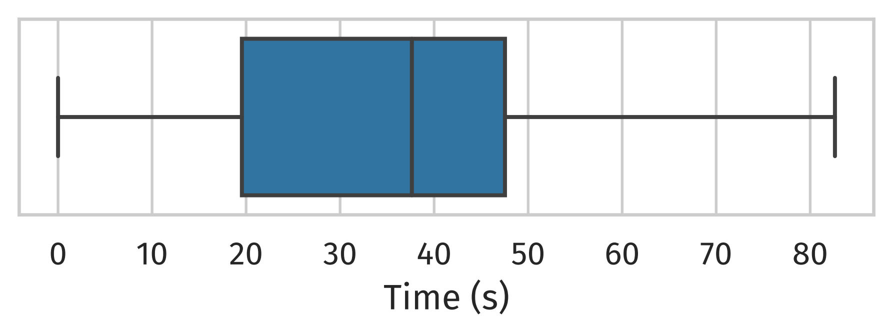

0
10
20
30
40
50
60
70
80
Time (s)
