# Supplementary material for: 3MSF: A Multi-Modal Adaptation of the 6TiSCH Minimal Scheduling Function for the Industrial IoT
Source: Sensors (Basel). 2024 Apr 10;24(8):2414. doi: 10.3390/s24082414 (PMC11054304; doi:10.3390/s24082414)
Supplement: Supplementary file 1 [file sensors-24-02414-s001.zip › data/plots/s2_stacked_area.pdf]

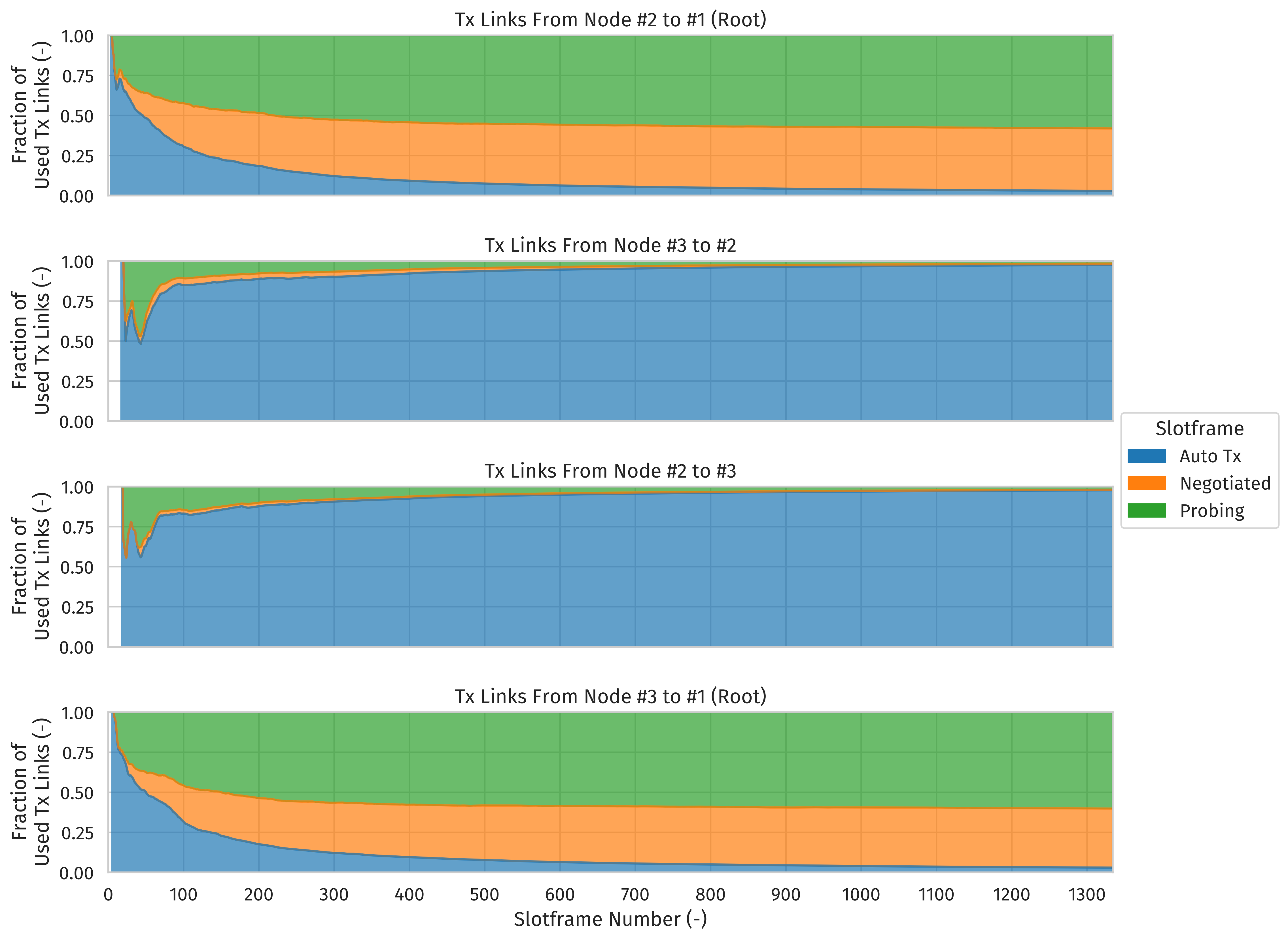

Tx Links From Node #2 to #1 (Root)
Tx Links From Node #3 to #2
Tx Links From Node #2 to #3
Tx Links From Node #3 to #1 (Root)
Fraction of Used Tx Links (-)
1.00
0.75
0.50
0.25
0.00
0
100
200
300
400
500
600
700
800
900
1000
1100
1200
1300
Slotframe Number (-)
Slotframe
Auto Tx
Negotiated
Probing
