# Supplementary material for: 3MSF: A Multi-Modal Adaptation of the 6TiSCH Minimal Scheduling Function for the Industrial IoT
Source: Sensors (Basel). 2024 Apr 10;24(8):2414. doi: 10.3390/s24082414 (PMC11054304; doi:10.3390/s24082414)
Supplement: Supplementary file 1 [file sensors-24-02414-s001.zip › data/plots/s3_parent_changes.pdf]

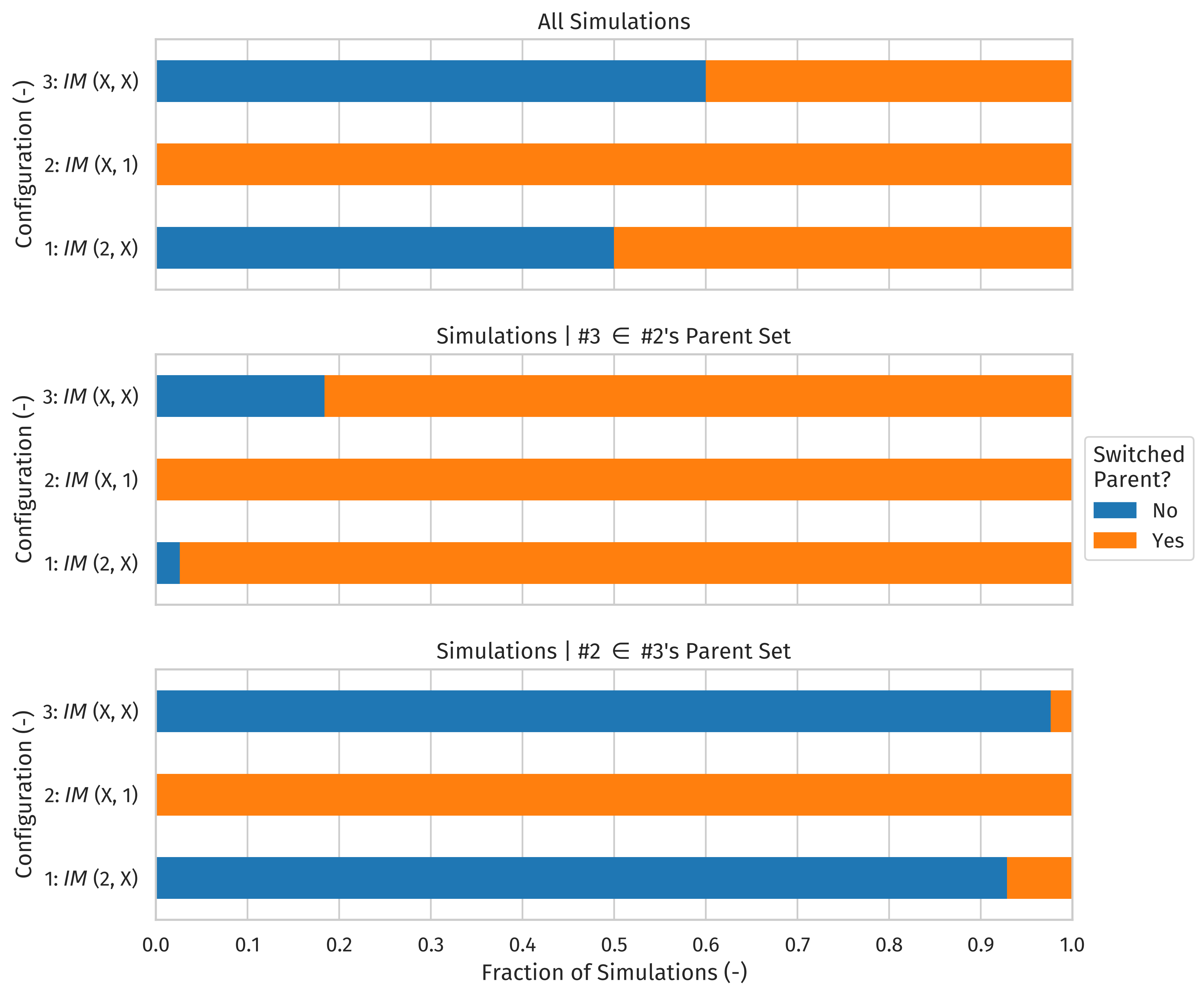

All Simulations
Simulations | #3 ∈ #2's Parent Set
Simulations | #2 ∈ #3's Parent Set
Configuration (-)
3: IM (X, X)
2: IM (X, 1)
1: IM (2, X)
0.0
0.1
0.2
0.3
0.4
0.5
0.6
0.7
0.8
0.9
1.0
Fraction of Simulations (-)
Switched Parent?
No
Yes
