# Supplementary material for: 3MSF: A Multi-Modal Adaptation of the 6TiSCH Minimal Scheduling Function for the Industrial IoT
Source: Sensors (Basel). 2024 Apr 10;24(8):2414. doi: 10.3390/s24082414 (PMC11054304; doi:10.3390/s24082414)
Supplement: Supplementary file 1 [file sensors-24-02414-s001.zip › navigation.pdf]

Multi-Modal Minimal Scheduling Function (3MSF)

This document describes how to navigate the supplementary files for the paper named "3MSF: A Multi-Modal Adaptation of the 6TiSCH Minimal Scheduling Function for Industrial IoT"

Table of Contents

- Multi-Modal Minimal Scheduling Function (3MSF)
  - Table of Contents
  - Directory Structure
    - code
      - contiki-ng
      - cooja
    - data
      - logs
      - plots
  - Recommended Resources

Directory Structure

The following sections have the same hierarchical structure as the supplementary files. While browsing these files on Windows is certainly possible, trying things out yourself will only work on Linux (preferrably Ubuntu 18.04 LTS or 20.04 LTS).

code

The directories contained in this folder are clones of the contiki-ng and cooja projects we modified for our research purposes. The file `instructions.pdf` contained in this folder explains how to use the contiki-ng project.

contiki-ng

This is a clone of the contiki-ng project. It is set to the `tdripl` git branch, which contains all our additions / modifications to contiki-ng. For more information, have a look at `Supplementary/code/instructions.pdf`. The twofaced example in `Supplementary/code/contiki-ng/examples/twofaced/` contains a lot of the important stuff, an so do the MSF service in `Supplementary/code/contiki-ng/os/services/msf/`, the 6top module in `Supplementary/code/contiki-ng/os/net/mac/tsch/sixtop/`, the RPL-Classic routing layer in `Supplementary/code/contiki-ng/os/net/routing/rpl-classic/`, the link statistics module `Supplementary/code/contiki-ng/os/net/link-stats.*`, and the cooja radio drivers `Supplementary/code/contiki-ng/arch/platform/cooja/dev/cooja-radio.*`. Note that contiki-ng is a Makefile-structured project primarily written in c.

cooja

This is a clone of the cooja project. It is set to the `dripl` git branch, which contains all our additions / modifications to cooja. For more information, have a look at `Supplementary/code/instructions.pdf`. Cooja is primarily written in Java. Note that cooja makes use of Java Native Interface (JNI) such that each node (or "cooja mote") executes an actual compiled Contiki-NG system by loading it as a native shared library. In short (if you look at the shared library as a state machine), each mote manipulates certain variables in memory (that is, the memory belonging to the Contiki-NG system attached to it), triggers all actions corresponding to one clock tick, and checks memory again for changes. Most of the action is centered around

`Supplementary/code/cooja/java/org/contikios/cooja/radiomediums/LogisticLoss.java` and `Supplementary/code/cooja/java/org/contikios/cooja/contikimote/interfaces/TwofacedRadio.java`.

data

This directory contains all the simulation data gathered during our simulation runs, in both raw and visualized format. For more information, have a look at `Supplementary/code/instructions.pdf`.

logs

This directory contains all the raw logs of the simulations we performed. More specifically, each log contains all the terminal output of the simulated nodes involved in a single experiment. By analyzing these log statements, a number of statistics may be derived. If you are following instructions (see `Supplementary/code/instructions.pdf`) on how to analyze raw log files with the appropriate python script, you can first copy the contents of this directory to `Supplementary/code/contiki-ng/examples/twofaced/logs/` such that you can perform log parsing without having to perform simulations (in order to get a feel for it first). Note that this directory is compressed in 7zip format to achieve a higher compression rate.

plots

Contains all graphs / plots generated from the respective simulation scenario in the corresponding directory (in pdf format).

Recommended Resources

- **The Contiki-NG Wiki**: It is generally advisable to start out here. However, the documentation is really basic and leaves much to be desired, especially if you want to do more than just write applications on top of existing functionality.
- **Jump Start Git** *by Shaumik Daityari*: As mentioned before, if you don't speak Git, this book is where you might want to start your journey.
- **C in a Nutshell** *by Peter Prinz & Tony Crawford*: Although C is an incredibly forgiving language when it comes to getting what you want out of it (hence the abundance of terribly written but otherwise "functional" code), some parts of our source code contain more advanced features and contstructs from the C99 and later C11 specification. We make no mistake about it, seasoned embedded developers would probably have a heart-attack looking at parts of our code, but the important thing to remember is that we're well on our way to write better source code and this book is what's getting us there.
- **The Java Programming Masterclass** *by Tim Buchalka*: want to learn modern Java programming with minimal to no experience in Object-Oriented Programming (OOP)? Just looking for a refresher on OOP best-practices and / or the Java programming language because it has been a while? Whatever your skill- and / or comfort level, this beautifully structured online course will get you there in no-time (provided you are willing to put in the work). Happy hunting!
